# Systematic over‐crediting in California's forest carbon offsets program

**DOI:** 10.1111/gcb.15943

**Published:** 2021-11-12

**Authors:** Grayson Badgley, Jeremy Freeman, Joseph J. Hamman, Barbara Haya, Anna T. Trugman, William R. L. Anderegg, Danny Cullenward

**Affiliations:** ^1^ Black Rock Forest Cornwall New York USA; ^2^ Lamont‐Doherty Earth Observatory Columbia University Palisades New York USA; ^3^ CarbonPlan San Francisco California USA; ^4^ National Center for Atmospheric Research Boulder Colorado USA; ^5^ Goldman School of Public Policy University of California, Berkeley Berkeley California USA; ^6^ Department of Geography University of California, Santa Barbara Santa Barbara California USA; ^7^ School of Biological Sciences University of Utah Salt Lake City Utah USA; ^8^ Stanford Law School Stanford California USA

**Keywords:** adverse selection, carbon offsets, climate policy, forests

## Abstract

Carbon offsets are widely used by individuals, corporations, and governments to mitigate their greenhouse gas emissions on the assumption that offsets reflect equivalent climate benefits achieved elsewhere. These climate‐equivalence claims depend on offsets providing real and additional climate benefits beyond what would have happened, counterfactually, without the offsets project. Here, we evaluate the design of California's prominent forest carbon offsets program and demonstrate that its climate‐equivalence claims fall far short on the basis of directly observable evidence. By design, California's program awards large volumes of offset credits to forest projects with carbon stocks that exceed regional averages. This paradigm allows for adverse selection, which could occur if project developers preferentially select forests that are ecologically distinct from unrepresentative regional averages. By digitizing and analyzing comprehensive offset project records alongside detailed forest inventory data, we provide direct evidence that comparing projects against coarse regional carbon averages has led to systematic over‐crediting of 30.0 million tCO_2_e (90% CI: 20.5–38.6 million tCO_2_e) or 29.4% of the credits we analyzed (90% CI: 20.1%–37.8%). These excess credits are worth an estimated $410 million (90% CI: $280–$528 million) at recent market prices. Rather than improve forest management to store additional carbon, California's forest offsets program creates incentives to generate offset credits that do not reflect real climate benefits.

## Graphical abstract

Forest carbon offsets are increasingly prominent in corporate and government “net zero” emission strategies, but face growing criticism about their efficacy. California's forest offsets program is frequently promoted as a high‐quality approach that improves on the failures of earlier efforts. Our analysis demonstrates, however, that substantial ecological and statistical shortcomings in the design of California's forest offset protocol generate offset credits that do not reflect real climate benefits.

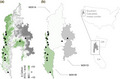

See commentary on: https://onlinelibrary.wiley.com/doi/10.1111/gcb.16008

## 1 INTRODUCTION

Carbon offset programs issue credits to projects that purport to avoid greenhouse gas emissions or remove carbon dioxide from the atmosphere. When policymakers allow polluters to use offset credits to comply with policy requirements, these “compliance offsets” increase the quantity of greenhouse gas emissions allowed within a legally binding policy regime in exchange for climate benefits claimed somewhere else (Cullenward & Victor, 2020; Erickson et al., 2014). For example, an oil refinery that is subject to an emissions limit might purchase an offset credit issued to a forest owner who agrees to reduce or delay timber harvest. The refinery can then claim the forest project's induced climate benefits to justify ongoing refinery emissions. Compliance offsets have been widely used in cap‐and‐trade programs in the European Union and California (Ellerman et al., 2016; Haya et al., 2020), as well as to satisfy climate mitigation pledges made under the Kyoto Protocol (Wara, 2007). The future use of offsets could include new national or subnational climate mitigation policies, along with international efforts under the United Nations Paris Agreement (Michaelowa et al., 2019; Schneider & La Hoz Theuer, 2019).

Offsets are also controversial. Because compliance offsets enable higher emissions within legally binding policy regimes, they must reflect real climate benefits that go beyond what is expected under counterfactual business‐as‐usual conditions—a standard called additionality (Bento et al., 2016). In the refinery example above, additionality requires that the polluter's purchase of carbon offsets causes new climate benefits. Although compliance offsets' additionality is a fundamental prerequisite to their successful inclusion in climate policy, this standard is not always achieved in practice (Gifford, 2020). Prominent studies concluded that the world's first major carbon offset programs, known as the Clean Development Mechanism and Joint Implementation, led to significant over‐crediting from projects that made suspect claims about the additionality of their efforts or the plausibility of their emissions under counterfactual baseline scenarios (Cames et al., 2016; Haya, 2010; Schneider & Kollmuss, 2015; Wara, 2007, 2008).

Because project‐specific claims are hard to evaluate and are easily exaggerated, some carbon offset programs, including the Clean Development Mechanism, shifted to a second‐generation or “standardized” approach to credit issuance. Under a standardized offsets paradigm, offset protocols set common rules for determining project eligibility, assigning projects' baseline scenarios, and calculating the number of credits that should be awarded to eligible activities (Hayashi & Michaelowa, 2013).

Although standardized offset protocol rules help avoid suspect project‐level claims observed in earlier programs, they also shift the risk of over‐crediting from project‐level claims to protocol‐level methodologies (Haya et al., 2020). One critical concern is the problem of adverse selection: because prospective offset project developers know more than regulators about likely project‐level baseline scenarios, they have an incentive to preferentially select projects that naturally outperform regulators' assumptions and therefore generate non‐additional credits (Bushnell, 2012; Fischer, 2005; Millard‐Ball, 2013; Montero, 1999). Thus, while a standardized protocol rule might prevent projects from customizing suspect methods to claim non‐additional credits, that same rule might also introduce bias or create perverse incentives for project developers. For example, a recent study identified systematic over‐crediting in Brazilian tropical forest offset projects, caused by standardized baselines that were unrepresentative of local forest conditions (West et al., 2020). Nevertheless, it is difficult to empirically analyze non‐additionality and other kinds of crediting errors because counterfactual scenarios are unobservable directly and can only be estimated indirectly through rigorous study with sufficient data and careful experimental design (Heilmayr et al., 2020; Montero, 1999; Schneider & Kollmuss, 2015).

Here, we identify a common statistical flaw, known as ecological fallacy, in the design of California's forest offsets program—the largest compliance market in active operation today. Ecological fallacy occurs when group‐level details, such as the mean of a distribution, are used to draw conclusions about individuals within that group. We show how this statistical problem leads to large‐scale over‐crediting. As explained further below, California's program awards the bulk of its offset credits to projects based on a comparison between projects’ initial measured carbon stocks and calculations of regional average carbon stocks. We evaluate whether these averages are in fact representative of the actual forests that participate in California's program by combining detailed information from public project documentation with data on forest carbon stocks from the U.S. Forest Service. Our approach allows us to directly and empirically estimate program crediting errors that are independent of concerns about the veracity of projects' unobservable counterfactual scenarios. As a result, our study offers a novel opportunity to empirically evaluate the performance of California's prominent compliance offsets market.

We begin with an introduction to California's forest offsets program to provide readers with the policy context needed to understand the ecological and statistical analysis at the core of our study. Next, we replicate the program's credit issuance calculations and show how a more ecologically robust definition of regional average carbon stocks reveals programmatic crediting errors. We then report over‐ and under‐crediting outcomes on a project‐by‐project and program‐wide basis. Finally, we close with a discussion of the crediting errors we identify—which represent only a subset of possible crediting errors—and their implications for the governance of carbon offset programs.

### 1.1 California's forest offset program

The California Air Resources Board (CARB) operates a cap‐and‐trade program that covers about 75% of statewide emissions and features a significant role for carbon offsets (Bushnell, 2012; Haya et al., 2020). Offset credits can be used by polluters subject to the cap‐and‐trade program to comply with their legal obligations, increasing the total amount of pollution allowed within the climate program in exchange for additional climate benefits calculated outside of it (Cullenward & Victor, 2020; Erickson et al., 2014). To earn offset credits, prospective offset projects must follow detailed rules and calculations in CARB's sector‐specific offset protocols, including third‐party verification of carbon measurements (Gifford, 2020; Haya et al., 2020). Projects submit applications to CARB, which checks that projects have correctly followed the applicable protocol rules and awards offset credits to qualified projects.

**FIGURE 1:**
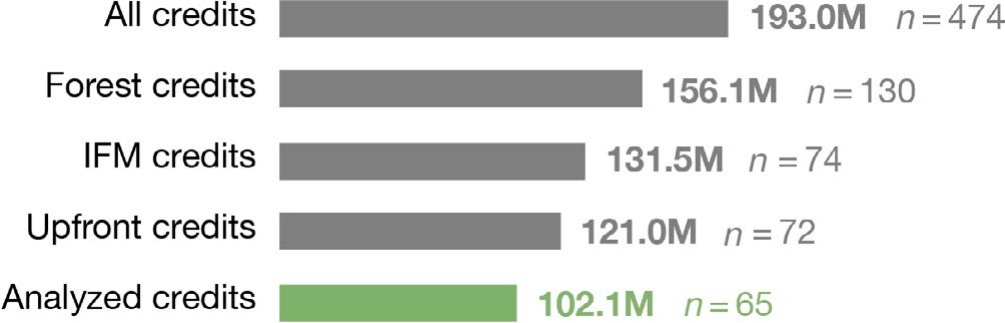
California's carbon offsets program. As of our study cutoff date of September 2020, the California Air Resources Board had issued 193 million offset credits, each worth 1 tCO_2_e, to 474 projects. The forest offsets protocol accounts for the vast majority of credits in the program, with most credits awarded to improved forest management (IFM) projects and most IFM credits earned in the form of initial, upfront credits calculated under standardized protocol rules. Limited public data disclosures restrict our analysis to 65 projects that earned 102.1 million upfront IFM credits, equivalent to about two‐thirds of the forest offsets program or about half of California's total offsets program

As of our study cutoff date of September 2020, CARB had issued about 193 million offset credits (each worth 1 tCO_2_e) across four compliance offset protocols (Figure 1; California Air Resources Board, 2020a). These credits represent a total value of about $2.6 billion at recent market prices of $13.67/tCO_2_e (California Air Resources Board, 2021). More than 80% of California's offset credits come from the U.S. Forest Projects protocol, which requires projects to use standardized baseline calculations when claiming credits from CARB (California Air Resources Board, 2011, 2014, 2015).

One striking feature of the forest offsets program is that the bulk of total offset credits are awarded to projects in their initial reporting period. Specifically, these credits are awarded to “improved forest management” (IFM) projects that reward management activities, like extended harvest rotations, that increase carbon stocks above modeled baseline scenarios (Kelly & Schmitz, 2016; Schmitz & Kelly, 2016). Our analysis exclusively focuses on these IFM projects and how they are awarded their initial tranche of offset credits, which we refer to as “upfront credits.” CARB had credited 74 compliance‐period IFM projects that encompass over 4 million acres of land as of our study cutoff date; upfront credits issued to these projects constituted about 63% of all offset credits in California's cap‐and‐trade program (Figure 1).

### 1.2 Upfront credits

Upfront credits are awarded to IFM projects based on the difference between initial on‐site carbon stocks, as measured by field surveys, and average carbon stocks in each project's projected baseline scenario over the next 100 years. The issuance of upfront credits depends on two key concepts.

First, California state law requires CARB to credit only “permanent” climate benefits. Although carbon dioxide from fossil fuel emissions remains in the atmosphere for hundreds to thousands of years (Archer et al., 2009) and has effects that are effectively permanent in duration (Pierrehumbert, 2014), CARB has defined a 100‐year period as demonstrating permanence and thus uses a 100‐year timeframe when calculating climate benefits under the forest offsets program (Ruseva et al., 2017). We retain this approach in our analysis for consistency.

Second, upfront payments help defray the initial financial costs of developing a carbon project (such as field surveys) and incentivize the protection of “carbon gems”—forested areas with especially high carbon stores (Anderson et al., 2017). Intuitively, upfront credits reward landowners who have higher‐than‐typical carbon stocks on their land and who claim that, in the absence of offset credits, they would harvest their forests according to their self‐reported baseline scenarios. Our analysis does not address the merits of upfront payments, which we take as given for the purpose of our study; instead, we seek only to analyze whether upfront payments cause real climate benefits.

**FIGURE 2:**
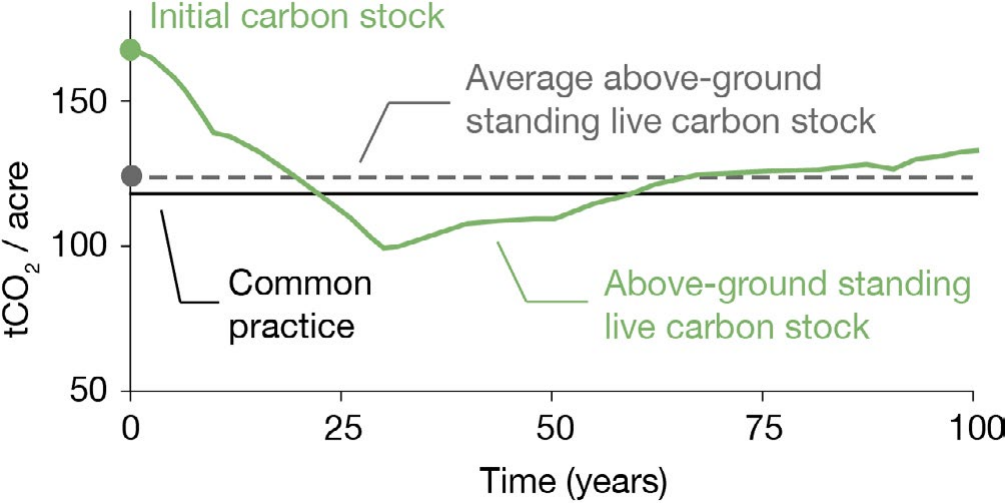
An example of a project baseline scenario. Initial carbon stocks (green dot) are determined via on‐site measurements. The baseline scenario (solid green line) represents a modeled scenario of how the project's forests could be managed in the absence of carbon offset payments. In this example, the baseline scenario depicts standing live carbon stocks declining from around 170 tCO_2_/acre to approximately 100 tCO_2_/acre after 25 years due to harvesting. The 100‐year average of carbon stocks in the baseline scenario is shown as a dashed grey line. Protocol rules require that average baseline carbon stocks (dashed grey line) must not fall below common practice (horizontal black line). Aside from this constraint, projects retain wide latitude in developing their baseline scenarios. The baseline scenario must be legally and financially feasible, but need not reflect typical, optimal, or even intended management activities. The example shown here is redrawn from the initial offset project data report of ACR324

Under protocol rules, projects cannot choose any baseline scenario they like. Projects must report baseline scenarios that forecast the effects of timber harvests and forest growth on carbon stocks in the absence of carbon payments (Figure 2). We focus exclusively on the 72 out of 74 projects in our sample for which initial carbon stocks exceed common practice—a regional measure of average carbon stocks calculated using U.S. Forest Service inventory data—because only these projects earn upfront credits. Although projects are generally free to model any baseline scenario that is legally and economically feasible, the 100‐year average of projects' modeled carbon stocks cannot fall below common practice. This rule prohibits projects from claiming they would harvest their forests below levels the protocol deems reasonable. In contrast to some voluntary forest offset programs, projects seeking credit in California's program cannot claim they would have simply razed their forest to the ground.

**FIGURE 3:**
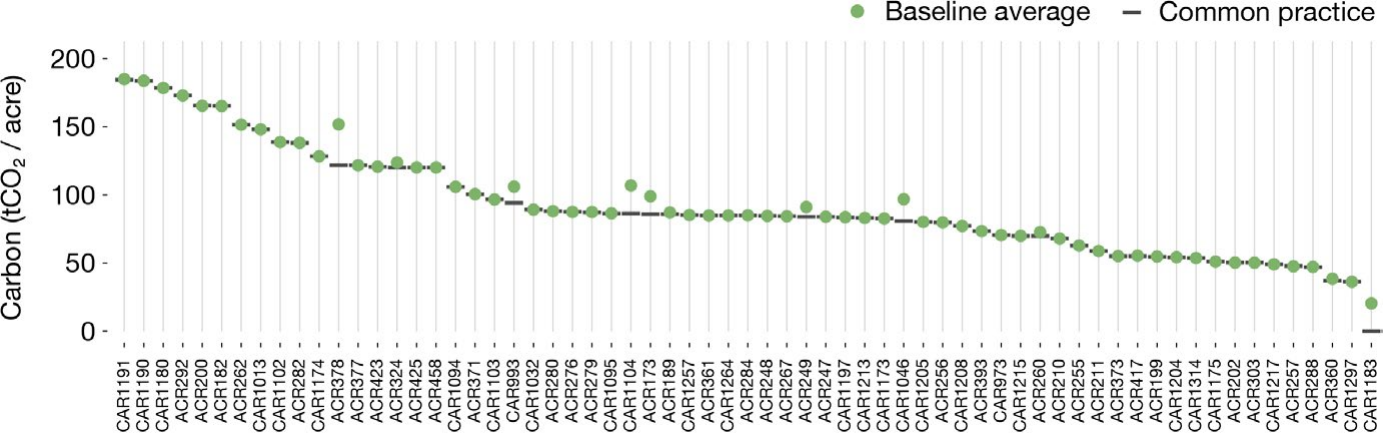
Forest carbon baseline scenarios converge to regional common practice estimates. Improved forest management (IFM) projects have baseline scenarios with 100‐year average carbon stocks that converge on protocol‐level calculations of regional common practice. The number of offset credits awarded to IFM projects depends on the difference between initial standing carbon stocks and the 100‐year average of carbon stocks in IFM projects' baseline scenarios, but these 100‐year averages are constrained by protocol rules to be no lower than regional estimates of common practice for similar forest types. For each project, the green circle shows carbon in projects' baseline scenario and the dark grey line shows common practice. 89% of projects analyzed are within 5% of common practice (mean ∆: 2.0 tCO_2_/acre, median ∆: 0.0 tCO_2_/acre)

CARB's decision to constrain projects' baseline scenarios using regional calculations of common practice is critical to determining the number of offset credits issued to projects. In practice, nearly all IFM projects' baseline scenarios converge to the most aggressive baseline harvest scenario allowed by protocol rules (i.e., down to common practice; Figure 3). This pattern maximizes the number of credits earned because upfront credits are awarded based on the difference between (1) a project's initial carbon stocks (which are determined by on‐site measurements) and (2) the 100‐year average carbon stocks in the project's baseline scenario (which are subject to discretionary modeling choices). Projects earn more credits the lower their claimed baseline scenario, which may explain why most projects choose baseline scenarios that converge on the lowest possible level allowed by protocol rules.

As a result of these two features—a protocol rule prohibiting IFM projects' 100‐year baselines from falling below common practice and the observed outcome that nearly all IFM projects report 100‐year baselines that converge toward or perfectly match common practice—the common practice numbers themselves are the primary determinant of upfront credits issued to IFM projects. In turn, because upfront credits to IFM projects constitute the dominant share of all forest offset credits issued as of our study cutoff date (121.0 million credits, or about 78%) and a majority of all the credits in California's entire offsets program (about 63%), the California regulator's choice of common practice is one of the most important factors determining program crediting outcomes. Understanding the method used to determine common practice is therefore critical to evaluating crediting errors in California's forest offsets program.

### 1.3 Calculating common practice

CARB calculates common practice using the U.S. Forest Service Forest Inventory and Analysis (FIA) database (Burrill et al., 2018), based on species combinations called assessment areas that span geographic regions known as supersections.

These two concepts—assessment areas and supersections—were initially developed by the Climate Action Reserve (Climate Action Reserve, 2010), a nonprofit carbon offsets registry, whose forest offsets protocol served as a template for CARB's program. To construct supersections, the Climate Action Reserve began with a set of eco‐topographic regions called ecosections that were developed by the U.S. Forest Service to define management areas with similar geology, climate, and vegetation communities (Cleland et al., 2007). These ecosections were combined to create novel supersections. Each supersection was then subdivided into one or more assessment areas representing species mixtures typical of forest types in that supersection. An example from the Northern California Coast supersection helps illustrate these concepts. Within this region of the United States, all tree species are assigned to one of two assessment areas: Redwood/Douglas Fir Mixed Conifer or Oak Woodland. Forest parcels dominated by oaks would be assigned to the Oak Woodland assessment area while those containing coniferous species would be classified as Mixed Conifer.

Having divided American forests into geographic regions (supersections) and species types (assessment areas), the Climate Action Reserve then established common practice for each assessment area using FIA data. Thus, every supersection has one or more assessment areas, and each assessment area has a common practice estimate of average carbon stocks derived from FIA data across that assessment area's supersection.

Although the Climate Action Reserve initially developed these methods for the voluntary offsets market, the California government subsequently adopted the same approach for compliance purposes in its cap‐and‐trade program. The California regulator, CARB, retained the same common practice numbers initially developed by the Climate Action Reserve in CARB's original 2011 U.S. Forest Projects protocol (California Air Resources Board, 2011) as well as in a 2014 update (California Air Resources Board, 2014). CARB subsequently worked with the U.S. Forest Service to update common practice numbers for the continental US and expand protocol eligibility to parts of Alaska (California Air Resources Board, 2015). Since the beginning of California's compliance offsets program through this writing, CARB has used the same IFM crediting methods described here.

## 2 METHODS

We quantify how the methods used to construct average carbon stocks affect crediting calculations. Drawing on a novel dataset digitized from public offsets project records, we directly estimate crediting errors in California's forest offsets program by comparing actual credits awarded against what would have been awarded using an ecologically grounded, project‐specific determination of common practice.

Instead of using a coarse average that combines ecologically distinct forest types to calculate average carbon stocks, we develop an ecologically grounded method to estimate a project‐specific common practice based on the species composition of each project and matching FIA plots. Specifically, we identify the forest types found in participating offsets projects from detailed project records and match these observational data to forest type codes used by the U.S. Forest Service using a classification algorithm. We then calculate an alternative estimate of average carbon stocks by sampling FIA plots in the same supersection that share the same forest type code, retaining the geographic supersection concept but replacing the protocol's coarse and unrepresentative assessment area construct with our species‐specific approach.

**FIGURE 4:**
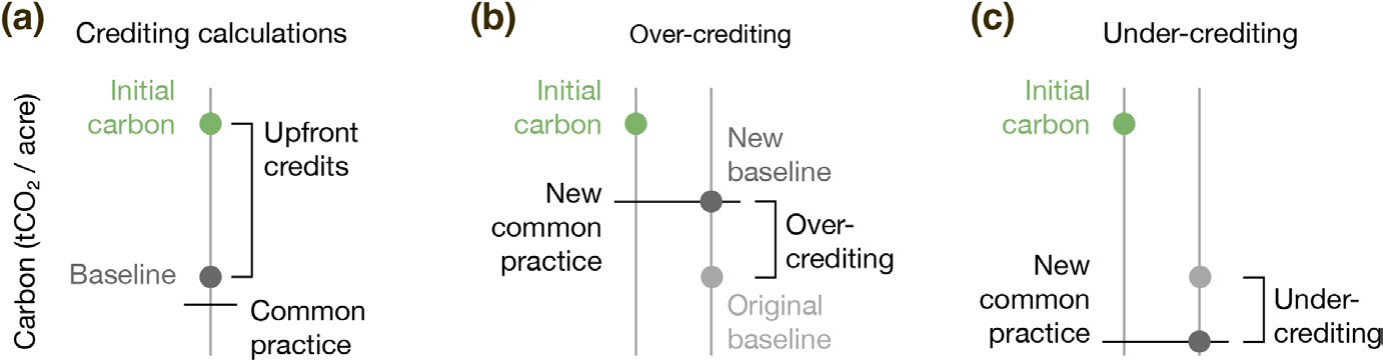
Upfront crediting methods. (a) Improved forest management (IFM) projects are awarded upfront credits based on the difference between projects' measured initial carbon stocks and the 100‐year average of carbon stocks in their projected baseline harvest scenarios. Under protocol rules, baseline averages must be equal to or greater than protocol‐defined common practice calculations. Our study estimates crediting errors by calculating a more ecologically appropriate common practice than is used in California's offsets program. (b) When our alternative common practice is higher than average carbon stocks in the baseline scenario, we report over‐crediting. (c) When our alternative common practice is lower than average carbon stocks in the baseline scenario, we report under‐crediting

Using the ecologically grounded calculation of average carbon stocks, we re‐calculate the number of credits projects would have received if project baselines had been constrained by our recalculated common practice estimates (Figure 4). Based on our recalculation, we report program‐wide crediting errors, including instances of over‐ and under‐crediting. We also identify statistical patterns of project development that indicate widespread adverse selection, by which project developers benefit from the statistical bias in a protocol that awards credits based on project‐level comparisons against coarse, regional baselines.

### 2.1 Offset crediting components

Upfront credits in forest offset projects are awarded based on the difference between a project's initial standing carbon and the 100‐year average of aboveground carbon in its baseline scenario. Common practice constrains the minimum carbon in that baseline scenario and is computed separately for each supersection and assessment area. Supersections are geographic regions comprised of multiple ECOMAP 2007 ecosections (Cleland et al., 2007). Assessment areas are groups of FIA forest types, each spanning a whole supersection, that are intended to reflect forest communities with similar ecological and economic attributes. Estimates of carbon from FIA are aggregated within each assessment area to derive common practice for that assessment area (California Air Resources Board, 2011, 2014, 2015). Our analysis evaluates whether these aggregations lead to offset crediting errors.

### 2.2 Digitized project records

We sourced project data from publicly available offset project data reports submitted to CARB (see Extended Methods), collecting all information necessary to replicate program credit issuance calculations (Figure S1), as well as the observed composition of tree species on participating project lands. We manually transcribed critical project attributes including total project acreage, initial carbon stocks, and the supersections and assessment areas involved in each project. We recorded 100‐year average standing live aboveground carbon stocks in project baseline scenarios. For projects' initial reporting period, we recorded onsite carbon stocks (denoted IFM‐1 and IFM‐3) and the carbon stocks contained within wood products (IFM‐7 and IFM‐8), both for the baseline and project scenarios, as well as the project's reported secondary effects and confidence deduction factors. We also transcribed all reported species with greater than 5% fractional basal area, on a per‐assessment‐area basis where data were available or else for the entire project. The schematized collection of records is available at https://doi.org/10.5281/zenodo.4630684.

### 2.3 Verification of crediting calculations

We verified the accuracy of our digitization by replicating actual project crediting calculations directly from project data using equation 5.1 from the 2015 CARB U.S. Forest Projects protocol (California Air Resources Board, 2015), which is identical to the method (equation 6.1) in the earlier 2011 and 2014 protocol versions (California Air Resources Board, 2011, 2014). Two members of our project team independently performed this exercise to ensure quality and converged on a unified result. We compared these estimates to the CARB‐reported project issuance table dated September 9th, 2020 (*R* ^2^ = 0.9998; Figure S1; California Air Resources Board, 2020a).

### 2.4 Forest inventory data

We analyzed data from the FIA database using rFIA, an open source software package that implements statistical practices recommended by the U.S. Forest Service (Bechtold et al., 2005; Stanke et al., 2020). We developed queries to estimate the total aboveground carbon and total acreage for every supersection, assessment area, site class, inventory period, and forest type, along with their variances. All subsequent estimates of common practice (either using CARB's approach or our alternative) sum carbon and acreage separately (after accounting for the appropriate expansion factors), before taking the ratio to report tCO_2_/acre (Stanke et al., 2020; Zarnoch & Bechtold, 2000).

### 2.5 Verification of common practice calculations

CARB's estimates of common practice aggregate carbon across all forest types within each assessment area and on a supersection‐wide basis. We confirmed that, from our processing of FIA data, we could independently reproduce CARB's common practice values by comparing our estimates directly to the values reported in the CARB‐provided Assessment Area Data File described in the forest offsets protocol and available on CARB's website (*R* ^2^ = 0.97, RMSE = 4.94 tCO_2_/acre; Figure S2; California Air Resources Board, 2015).

### 2.6 Alternative species‐specific common practice

We developed an alternative, more ecologically robust definition of common practice using project‐reported species composition data. We compare each project against a project‐specific (and therefore more representative) subset of FIA data, as opposed to the coarse regional averages of the CARB protocol. We built a classification algorithm to map species composition (as reported in publicly filed project paperwork) to forest types (a set of canonical species groupings reported by FIA). We then fit a radius‐neighbors classifier using pairs of two reported quantities in the FIA database: fractional basal area per species (derived from per‐tree measurements) and recorded forest type code. Both quantities—tree species and forest type—are reported on a “per‐condition” basis, where all FIA measurement plots are assigned to one or more “condition classes.” Each condition is assigned a single forest type. Combined, these data allow us to translate species prevalence to forest type codes. For every project, the classifier returns a list of forest types and the probability that the project belongs to those forest types. We then use these forest type assignments to estimate common practice from FIA plots with those forest type codes.

Intuitively, the classifier takes species composition data as an input and estimates the probability of that species mixture belonging to each FIA‐defined forest type based on relative similarity to the species composition of FIA plots. We fit a separate classifier for each supersection based on all FIA plots within the supersection boundaries. We used grid search and five‐fold cross‐validation to find the radius (“neighborhood”) that maximized the classifier's ability to predict FIA‐reported forest types from FIA‐observed species data. The median, weighted F1 accuracy score (which considers Type I and Type II classification errors) across all classifiers was 0.78, with 1 being the best score (Table S1).

### 2.7 Calculation of crediting errors

We used our alternative species‐specific common practice to calculate a new 100‐year average of carbon stocks in each project's baseline scenario, assuming that projects would have selected new baseline scenarios such that the new common practice estimate would constrain average baseline carbon stocks. Rather than simply replace the common practice reported by the project with our estimate, however, we scale a project's reported common practice by the assessment‐area‐weighted ratio of our alternative calculation of common practice to our own re‐calculation of CARB's assessment area estimates (Figure S2). Scaling by this ratio ensures that changes in common practice are due exclusively to changing assumptions about how FIA data are aggregated, not any issues with our ability to reproduce the original common practice numbers used in CARB's protocol (see Extended Methods). These steps allow us to estimate the credits that would have been awarded to actual projects using our alternative common practice calculation. We obtained confidence bounds on our estimates of crediting error through Monte Carlo error propagation. Using variances of carbon per acre from FIA for each forest type and assuming Gaussian noise, we sampled 1000 random draws of FIA carbon estimates and on each draw calculated the crediting error for individual projects. Throughout, we report the 5th, 50th, and 95th percentiles of the resulting distributions.

## 3 RESULTS

**FIGURE 5:**
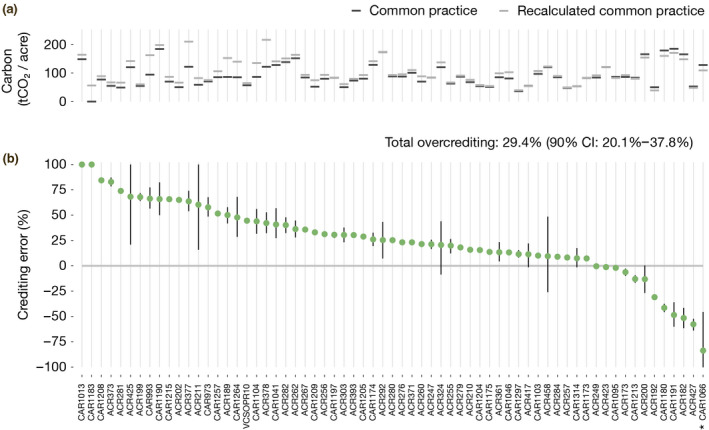
Estimated crediting error by project. We re‐calculate the number of credits that would have been awarded to forest offset projects with a more ecologically robust measure of common practice. (a) The difference between common practice numbers used under protocol rules (dark grey lines) and our more ecologically robust common practice numbers (light grey lines) for each project. Over‐crediting occurs when our common practice calculation estimate produces more carbon per acre compared to California Air Resources Board's common practice values, and under‐crediting occurs when our common practice estimate results in less carbon per acre. (b) The extent of over‐ and under‐crediting as a percentage of actual credits awarded to each project. Green circles indicate each project's median estimate for over‐ or under‐crediting, with vertical black lines spanning the 25th and 75th percentile estimates. Across the population of projects analyzed, total over‐crediting is estimated at 30.0 million tCO_2_e (90% CI: 20.5–38.6 million tCO_2_e) or 29.4% of the credits we analyzed (90% CI: 20.1%–37.8%; *Note that the bottom of the confidence interval for CAR1066 is truncated)

For the vast majority of IFM projects receiving upfront credits, our more ecologically robust estimate of common practice is higher than the supersection‐wide values used in the California forest offsets program, which implies over‐crediting. For a minority of projects, we find a lower common practice, which implies under‐crediting (Figure 5).

To illustrate our results and make causal factors concrete, we first describe detailed results for three representative projects that our analysis indicates were over‐credited (identified by their registry numbers ACR189, ACR361, and CAR1183). Next, we do the same for three projects that our analysis indicates were under‐credited (ACR200, CAR1180, CAR1191). We then report aggregate statistics that identify net over‐crediting across the program as a whole.

Perhaps the most important example of over‐crediting occurs in the Southern Cascades supersection, which ranges from the Pacific coast to the foothills of the Sierra Nevada and hosts the most offset projects of any supersection in California's program. Within this region, CARB protocol rules specify that temperate, carbon‐dense forest types like Douglas Fir (*Pseudotsuga menziesii*; average 122.5 tCO_2_e/acre) and tanoak (*Notholithocarpus densiflorus*; average 192.4 tCO_2_e/acre) are averaged together with less‐carbon‐dense forest types that occupy more arid niches, like ponderosa pine (*Pinus ponderosa*; average 60.4 tCO_2_e/acre). Comparing project carbon against this amalgamation of wet and arid forests causes projects like ACR189, which is composed primarily of Douglas fir (26% of basal area) and tanoak (49% of basal area), to receive substantial upfront credits under protocol rules simply due to a mismatch between the species in the project and the species included in the regional average. By instead comparing ACR189 against FIA plots that contain primarily Douglas fir and tanoak, a more ecologically robust comparison, we estimate that ACR189 is over‐credited by 135,869 tCO_2_e (90% CI: 85,481–185,917 tCO_2_e) or 50.1% of its total credits (90% CI: 31.5%–68.6%).

Similar dynamics play out in the temperate rainforests of coastal Alaska, where orographically induced precipitation and relatively warmer oceanside temperatures allow charismatic species like Sitka spruce (*Picea sitchensis*; average 121.1 tCO_2_e/acre) and western hemlock (*Tsuga heterophylla*; average 143.0 tCO_2_e/acre) to accumulate massive stores of carbon (Keith et al., 2009). ACR361, for example, consists of 94.9% Sitka spruce by basal area. Yet, the common practice against which this Sitka‐dominated forest is compared contains carbon estimates from far‐less‐carbon‐dense forest types like cottonwood (*Populus* spp.; average 41.4 tCO_2_e/acre) and paper birch (*Betula papyrifera*; average 38.3 tCO_2_e/acre). Comparing ACR361 instead against other Sitka spruce forests from FIA measurements across the full coastal Alaska region indicates median over‐crediting of 318,269 tCO_2_e (90% CI: −198,607 to 871,385 tCO_2_e) or 13.4% of its total credits (90% CI: −8.4% to 36.7%).

The most surprising example concerns a mixed conifer project, CAR1183, in the “sky island” forests of New Mexico (DeBano et al., 1995). Despite the project consisting primarily of Douglas fir (37.1% of basal area) and ponderosa pine (22.9% of basal area), the rules of the offsets protocol allowed CAR1183 to enroll itself under the Pinyon/Juniper Woodland assessment area. Perplexingly, in the 2011 and 2014 versions of California's protocol, this assessment area had a common practice of 0 tCO_2_/acre (California Air Resources Board, 2011, 2014). Though CARB would later update this number to 8.74 tCO_2_/acre in its 2015 protocol (California Air Resources Board, 2015), CAR1183 was developed under the earlier rules and earned 4.4 million upfront credits. In fact, under the earlier rules, any forest in that region would have been eligible for upfront credits. When more appropriately compared to FIA plots that contain Douglas fir and ponderosa pine, CAR1183's initial carbon stocks fall below the regional average. As a result, we estimate that 100% of the project's claimed emission reductions are over‐credited, a result that is robust across the full 5%–95% confidence interval.

We also identify a handful of projects as under‐credited. Several of these projects, such as ACR200, CAR1180, and CAR1191, occur in the Northern California Coast supersection, a region characterized by iconic and commercially valuable Douglas fir and redwood (*Sequoia sempervirens*) forests. Notably, tanoak trees are among the most common species (by basal area) in each of these under‐credited projects. This causes our classifier to characterize these forests as “Tanoak,” a forest type with an average standing live biomass of 173.7 tCO_2_e/acre, as opposed to the Northern California Coast Redwood/Douglas‐fir Mixed Conifer assessment area common practice of 205.15 tCO_2_e/acre. Because our reclassification lowers the projects' common practice, our approach would have allowed projects to claim a more aggressive baseline harvest scenario than they actually did—and therefore we report these projects as under‐credited (Figure 4c). Interestingly, the dominance of tanoak can arise as a legacy effect caused by the preferential harvest of commercially valuable conifers (Waring & O'Hara, 2008). Though these forests are predominantly tanoak today, that balance will likely shift over the coming decades while more merchantable Douglas fir and redwood trees grow to maturity. Our classifier evaluates the mix of species as they exist today and does not account for expected long‐term changes in species composition. Doing so would likely pose significant methodological challenges but would also more accurately capture long‐run forest carbon cycle dynamics and, at least in these three cases, reduce the extent of reported under‐crediting.

Across the program as a whole, we find evidence of systematic over‐crediting (Figure 5). Of the 102.1 million tCO_2_e worth of upfront credits for which we have sufficient data to analyze, we estimate net over‐crediting of 30.0 million tCO_2_e (90% CI: 20.5–38.6 million tCO_2_e) or 29.4% of the credits we analyzed (90% CI: 20.1%–37.8%). At recent market prices of $13.67 per offset credit (California Air Resources Board, 2021), these excess credits are worth $410 million (90% CI: $280–$528 million)—and likely more, as market prices could rise if CARB took steps to correct for over‐crediting.

Uncertainty ranges in our project‐specific and program‐wide results reflect uncertainty in the underlying U.S. Forest Service FIA data. Although CARB uses point estimates of common practice, all calculations based on FIA data are subject to sampling uncertainty. As indicated in Figure 5, some project‐level estimates of crediting error have large confidence intervals (e.g., ACR211, ACR458), whereas others have narrow intervals (e.g., CAR1215, ACR260). The differences typically reflect the number of matching FIA plots in the project's supersection (see Extended Methods). Some locations have relatively few plots, which leads to higher uncertainties in estimates of common practice—notably in Alaska, where FIA sampling is sparse.

## 4 DISCUSSION

### 4.1 Statistical bias in geographic regions

The fundamental challenge with awarding upfront offset credits via standardized protocol rules lies in defining an ecologically robust point of comparison. The California forest offsets protocol aggregates FIA data across assessment areas (species types) and supersections (geographic regions). We identify statistical patterns of project development that indicate widespread adverse selection. We find that projects are preferentially located in forests where carbon stocks naturally exceed coarse, regional averages, and thus generate substantial credits that do not provide real and additional climate mitigation benefits.

**FIGURE 6:**
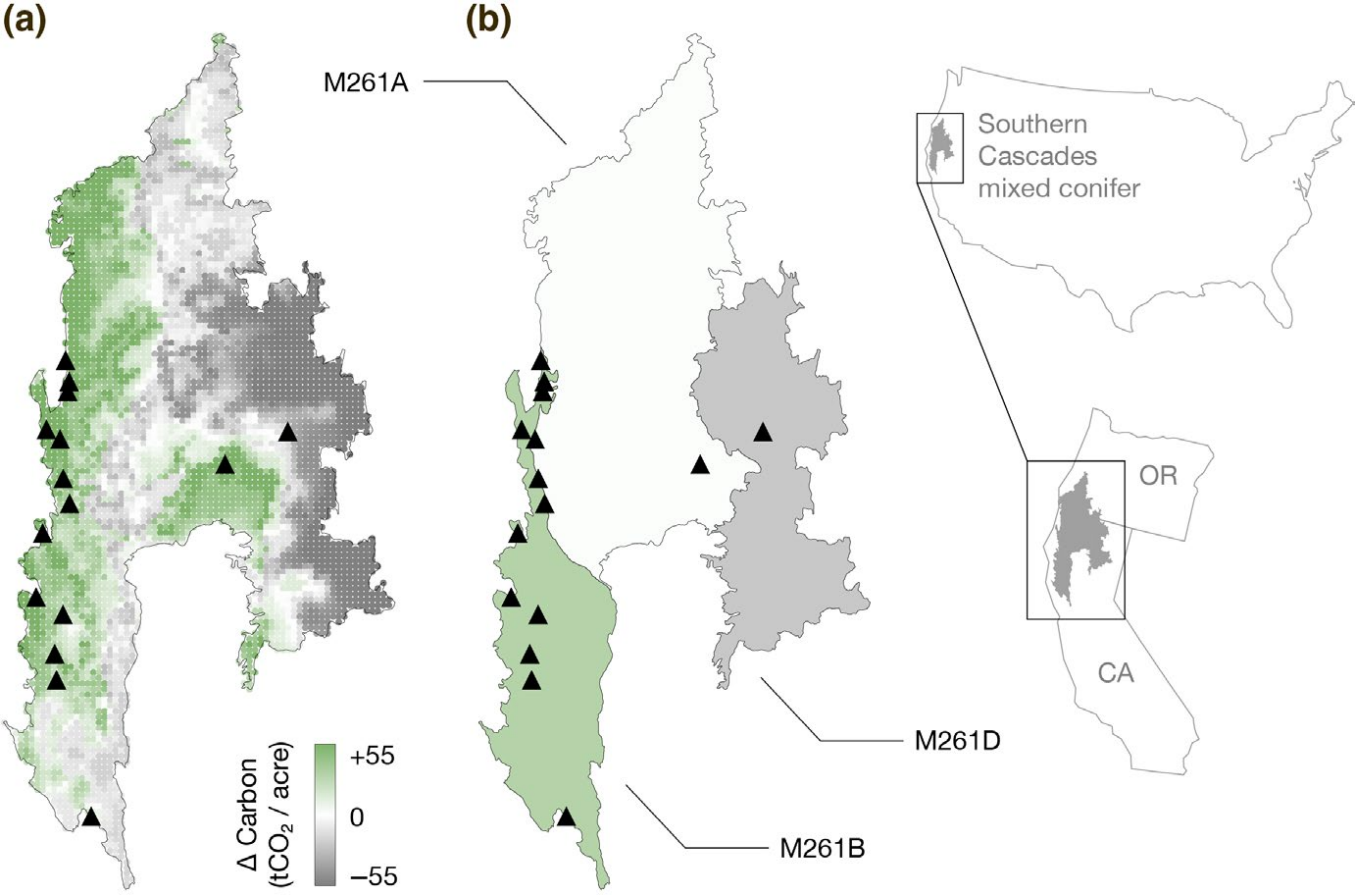
Arbitrage patterns in the Southern Cascades mixed conifer assessment area. One of the most extreme examples of over‐crediting occurs in the mixed conifer assessment area of the Southern Cascades supersection. (a) The difference between standing live aboveground forest carbon in FIA plots that are climatologically similar to local conditions, and the supersection‐wide average of all plots (see Extended Methods). Projects, represented with black triangles, cluster in carbon‐rich areas, notably in wetter climates near the coast where carbon‐dense forests grow. (b) The difference between ecosection‐ and supersection‐wide common practice for mixed conifers. Three ecosections with distinct local carbon patterns were combined together to generate a supersection‐wide common practice number that distorts ecological reality. The most carbon‐rich ecosection (M261B) contains most of this supersection's offset projects, which earn credits based on comparisons against supersection‐wide averages that include dryer and less temperate ecosections (M261A, M261D)

Part of the problem involves the construction of supersections. The mixed conifer assessment area in the Southern Cascades supersection, which hosts more projects than any other supersection, provides a powerful illustration (Figure 6). The supersection is composed of three smaller U.S. Forest Service ecosections. Starting on the supersection's western edge, ecosection M261B features relatively wet, carbon‐dense forests with average carbon stocks for mixed conifers forest types of 150.5 tCO_2_/acre. But this ecosection is combined with two others, M261A and M261D, that have drier and less‐carbon‐dense forests (120.6 and 100.6 tCO_2_/acre, respectively). Under CARB's protocol rules, the supersection‐wide common practice for mixed conifer forests is 121.8 tCO_2_/acre, which makes an “average” forest in M261B immediately eligible for upfront credits. Although CARB claims that combining ecosections with substantially different average carbon stocks does not change regional common practice by more than 10% (California Air Resources Board, 2011, 2014; Climate Action Reserve, 2010), the creation of the Southern Cascades supersection appears to have violated this condition: the protocol's 121.8 tCO_2_/acre is a −19% change from the M261B average of 150.5 tCO_2_/acre. Figure 6 shows clear clustering of projects within M261B, all of which likely benefit from the ecologically suspect combination of ecosections.

Although the Southern Cascades supersection is an extreme example, using any form of geographic aggregation risks a specific type of ecological fallacy known as the modifiable areal unit problem (Gehlke & Biehl, 1934). Simple averaging over underlying variations in climate and its relationship to carbon storage necessarily introduces opportunities for adverse selection (Figure 6a). Biogeographers have long understood the challenge of drawing firm boundaries around ecological regions or categories of species because while boundaries help communicate with outside audiences, border regions are complex areas where the characteristics of separate regions interact (Bailey, 2004; Omernik, 2004; Omernik & Griffith, 2014). When used, spatial aggregation should be adopted carefully on the basis of ecologically meaningful boundaries and stress‐tested for the potential to encourage adverse selection. We report results based on the same supersection boundaries used in the California protocol for the sake of comparability, not as an endorsement of this approach.

### 4.2 Data limitations

Moving to species‐specific analysis, such as our alternative approach to calculating common practice, partially addresses but does not completely avoid statistical challenges to a precise definition of common practice. Areas of the United States with extensive FIA sampling support common practice comparisons that are better grounded in ecology. But in other regions, notably Alaska, limited sampling is a barrier to robust estimates of common practice. For example, the Alaska assessment area “North Coast Mountains, Chugach‐St. Elias Mountains and Gulf of Alaska” has a mere 79 FIA plots, which serve as the basis for issuing over 9.5 million upfront credits. By contrast, the “Southern Cascades mixed conifer” assessment area in California and Oregon has upwards of 500 FIA plots.

Adopting a species‐specific approach for calculating common practice necessarily reduces the number of FIA plots available for comparison in exchange for improved ecological rigor. However, it is not automatically the case that having fewer plots results in greater uncertainty in common practice estimates. Uncertainty is a function of both sample size (which gets smaller with finer‐resolution comparisons, thereby increasing uncertainty) and the variation in measured carbon at those FIA plots (which can also get smaller with narrower comparisons, thereby potentially decreasing uncertainty). Aggregating over larger geographic areas increases sample size, but does not necessarily produce more accurate (i.e., less biased) carbon estimates. In fact, mixing dissimilar tree species and forest types decreases accuracy and results in a well‐described logical fallacy known as ecological fallacy (Gehlke & Biehl, 1934).

Our analysis addresses the tradeoffs inherent with smaller sample sizes and the existence of within‐species variability by explicitly accounting for variance in estimated carbon stocks across both species and space. In contrast, California's forest offsets protocol does not explicitly account for uncertainty. We suggest that it is not enough to simply invoke the use of FIA data to assert the quality of forest carbon estimates; a reliable protocol must also show how sampling density and statistical uncertainty are managed through rigorous protocol design (Bento et al., 2016; Haya et al., 2020).

Finally, we note that limitations in projects' reported data on species composition affect our ability to precisely quantify crediting errors. Though the majority of projects report species composition on a per‐assessment‐area basis, some projects only report species composition for the project as a whole (i.e., across multiple assessment areas). In these instances, we assume that project‐level reporting data applies uniformly across all project lands. This assumption directly translates into uncertainty in forest type classification, a critical step in our analysis of crediting errors. As demonstrated in our discussion of project under‐crediting of tanoak‐dominated forests in transition, it is also the case that species composition data are not always reflective of a project's “true” (or “terminal”) forest type. Higher resolution species data, either reported by the project or perhaps gathered from satellite remote sensing, could help refine crediting error estimates.

### 4.3 Baseline patterns and non‐additionality

A key feature of our study is that it does not depend on counterfactual analysis to critique additionality claims. Claims that entire projects are non‐additional are important to consider but difficult to evaluate quantitatively because counterfactual scenarios cannot be observed. In contrast, our analysis uses revealed program outcomes to directly estimate a specific subset of crediting errors. Nevertheless, the observation that nearly all offset projects choose baseline scenarios that converge on common practice (Figure 3) raises broader additionality concerns. It is possible that some projects' “true” baseline scenarios would be lower than protocol rules allow, such that converging on common practice would be appropriate for these projects. However, it is implausible that nearly all projects are in this situation, particularly since our re‐estimate of common practice tends to be higher, not lower, than what the California program assumes. We also found evidence that projects specifically target common practice in baseline modeling. As one example, ACR373's project documentation explains how linear optimization was used to drive the project's baseline scenario as close to common practice as possible. Finally, we note that baseline over‐crediting can be carefully combined with other estimates of over‐crediting, such as extrinsic evidence that an entire project is non‐additional or estimates of market‐wide emission leakage effects, but we do not attempt that here.

### 4.4 Over‐crediting and the California buffer pool

California law requires that offsets be “real, permanent, quantifiable, verifiable, and enforceable” (California Health & Safety Code § 38562(d)(1)) and that project baselines reflect “a conservative estimate of business‐as‐usual” conditions (California Code of Regulations, title 17, § 95972(a)(3)). We estimate baseline over‐crediting of 30.0 million tCO_2_e (90% CI: 20.5–38.6 million tCO_2_e). One additional step is needed to evaluate the climate‐equivalence claim made by California's offsets program. The California forest protocol features a buffer pool, into which forest projects contribute a share of their total credits (up to about 20%; California Air Resources Board, 2020b). The purpose of the buffer pool is to protect against risks to forest carbon from factors such as fire, drought, and bankruptcy to ensure that forest carbon is stored for a 100‐year permanence period; however, credits in the buffer pool can, in theory, be used to compensate for any environmental inadequacy in the program. Our results indicate that over‐crediting is likely larger than the program's buffer pool, which contained 24.6 million tCO_2_e as of October 2020 (California Air Resources Board, 2020b). Even if over‐crediting occurs at only the 5th percentile of our estimate (20.5 million tCO_2_e), addressing the environmental integrity of that outcome would deplete 83% of the buffer pool, leaving it severely undercapitalized in the face of growing climate risks (Anderegg et al., 2020; Herbert et al., 2020). This result calls into question whether California's offsets program achieves the state's policy goals.

## 5 CONCLUSION

We quantify systematic statistical and ecological shortcomings in California's forest offsets protocol, which issues upfront carbon credits to IFM projects on the basis of flawed calculations of average regional carbon stocks. At its core, our analysis demonstrates how averaging together dissimilar tree species across arbitrarily defined geographic regions allows—and, via adverse selection, may even encourage—offset projects that claim spurious, non‐real carbon credits. Our median results indicate that nearly a third of credits we analyzed do not reflect real climate benefits and are, instead, the consequence of methodological shortcomings. Our results only describe a subset of possible crediting errors, however, and do not address separate concerns about non‐additional projects, emissions leakage, or forest carbon permanence.

Though our analysis quantifies one important type of over‐crediting in California's forest offsets program, identifying the problem is far easier than resolving it. Constructing a comparable sample of FIA plots that share similar environmental conditions and ownership histories with all eligible forest parcels requires careful consideration that goes beyond the work presented here. Some eligible forests might lack truly representative points of comparison in the FIA database; others might have some comparable FIA plots, but not enough to provide adequate statistical certainty to justify upfront credit issuance. California's program errs on the side of making more forests eligible at the expense of constructing unrepresentative averages. As a result, the program allows for significant crediting of non‐real, non‐additional climate benefits. Although our alternative method aims to construct better averages, it could require the exclusion of forest types or geographies that lack sufficient public data to avoid the errors we document here. Nevertheless, our methods provide a reasonable basis for estimating programmatic crediting errors. Not only do they improve on key ecological shortcomings in current protocol rules, but they are also independent of the effects of adverse selection: because our methods were not used to issue real‐world credits, they generate an unbiased estimate of crediting errors that follow from offset project developers responding to the incentives provided by current program rules.

Finally, our results highlight the importance of carbon offsets governance. The statistical errors underlying California's forest offsets program provide a stark example of the risks inherent in the standardized approach to issuing carbon credits. In pursuit of the laudable goals of broad eligibility and uniform methods, California's rules generate widespread opportunities for adverse selection because local project conditions can naturally diverge from regional averages. Avoiding over‐crediting requires not only anticipating these problems in the offsets protocol design phase—such as through a more granular analysis of average carbon stocks across species and geographies, explicit credit issuance discount factors that account for expected adverse selection outcomes, and/or limiting protocol eligibility conditions—but also an active monitoring strategy to detect problematic outcomes early, before tens of millions of credits are affected. We also suggest policymakers consider incorporating program evaluations conducted by financially disinterested parties. In contrast, a governance regime that focuses primarily on protocol design remains vulnerable to unanticipated problems that can subsequently be exploited by actors who need only follow the rules.

## CONFLICT OF INTEREST

The authors declare no conflicts, financial or otherwise, that could be perceived as influencing the research described here. D.C. is the Vice Chair of California's Independent Emissions Market Advisory Committee but does not speak for the Committee here.

## AUTHORS’ CONTRIBUTIONS

Grayson Badgley, Danny Cullenward, Jeremy Freeman, and Joseph J. Hamman designed the research; Grayson Badgley digitized the project report data; Grayson Badgley, Danny Cullenward, Jeremy Freeman, Joseph J. Hamman, and Barbara Haya performed the research and analyzed the data; all authors contributed to interpreting the results and writing the paper.

## Supporting information

Supplementary Material

## Data Availability

The source code to reproduce our analysis is available in https://doi.org/10.5281/zenodo.5567962 and https://doi.org/10.5281/zenodo.5567957. Archival versions of this project's data products are available in https://doi.org/10.5281/zenodo.4630684 and https://doi.org/10.5281/zenodo.5565457.
