# Supplementary material for: Systematic over‐crediting in California's forest carbon offsets program
Source: Glob Chang Biol. 2021 Nov 12;28(4):1433–45. doi: 10.1111/gcb.15943 (PMC9299598; doi:10.1111/gcb.15943)
Supplement: Supplementary file 1 — Supplementary Material [file GCB-28-1433-s001.pdf]

**Supplementary Methods**

# Systematic over-crediting in California's forest carbon offsets program

Grayson Badgley*, Jeremy Freeman, Joseph J. Hamman, Barbara Haya, Anna T. Trugman, William R.L. Anderegg, Danny Cullenward*
* graysonbadgley@gmail.com; danny@carbonplan.org

Here we present key sections of our methods in more detail. Section names below correspond to matching sections of the Brief Methods in the primary article.

## Contents

**Digitized project records** 2
**Verification of crediting calculations** 5
**Forest inventory data** 6
**Verification of common practice** 7
**Classification algorithms** 9
**Calculation of under- and over-crediting** 11
**Spatial arbitrage patterns** 16
**Open source software and data** 17
**References** 18
**Glossary** 19
**Appendix 1: Verification of crediting calculations** 23
**Appendix 2: Classification labels** 28

**Digitized project records**

We sourced project data from project-submitted “offset project data reports” (OPDRs), the official documentation offset projects submit to CARB. These documents are made available by the three offset project registries that help CARB administer California’s offset program: the Climate Action Reserve (CAR), the American Carbon Registry (ACR), and Verra (VCS).

For each project, we transcribed project details described in the “initial” and “annual” OPDRs. In the rare case where initial and/or annual OPDRs were unavailable, we sourced information from the project’s listing information (which is also hosted by the offset registries), taking note of the discrepancy. We recorded critical project attributes such as total project acreage, reported initial carbon stocks, and the supersections and assessment areas of each project. For project baseline scenarios, we recorded the 100-year average standing live aboveground carbon stock. In most cases, this variable was directly reported in the text of the initial OPDR and its supplements. However, in some cases, only a graphical depiction of the baseline scenario was provided. In these cases, we used a graph digitization tool to infer 100-year average standing live aboveground carbon. In some cases, when both common practice and the 100-year average standing live aboveground carbon stocks were both clearly displayed and were visually indistinguishable, we recorded the 100-year average standing live aboveground carbon stock as being equal to common practice.

For the initial reporting period, we recorded onsite carbon stocks (denoted IFM-1 and IFM-3) and the carbon stocks contained within wood products (IFM-7 and IFM-8), both for the baseline and project scenarios as well as for the project’s reported secondary effects. Onsite carbon stocks (IFM-1 and IFM-3) for the “project scenario” were further adjusted by the project-reported confidence deduction factor that reduces projects’ earned offset credits due to statistical uncertainty in on-site carbon measurements above a 5% threshold (1). These data allowed us to recalculate the number of ARBOCs that should have been granted to the project based on publicly available documents for each projects’ first reporting period.

When reported, we also transcribed details about the species composition of each project. As detailed in Section 3.1(a)(1) of the 2015 US Forest Projects protocol, projects must report the species makeup of each individual assessment area in terms of fractional basal area, which is then compared against an assessment area specific “Species Diversity Index” that is reported alongside common practice numbers in the CARB-provided Assessment Area Data File. We recorded all species, on a per assessment area basis, with greater than 5% fractional basal area. Some projects only reported species composition on a whole-project basis, which we recorded and, in subsequent analyses, assumed all assessment areas had that same, fixed species composition (see below). All species were denoted using the appropriate FIA species code from Appendix F of the FIA User Guide (2).

While not used directly in our analysis except for plotting purposes, shapefiles for projects were obtained from the California Air Resources Board's online Credit Issuance Map, accessed at https://webmaps.arb.ca.gov/ARBOCIssuanceMap/ using the ArcGIS MapServer API. An archival copy of standardized and processed shapefiles as GeoJSON is available at http://doi.org/10.5281/zenodo.4630684.

Our full digitized database can be downloaded in JSON and CSV formats. Most of the associated metadata, alongside a subset of our analysis results, can be browsed in an interactive map, all available at https://carbonplan.org/research/forest-offsets. A sample project record from the database is shown in Supplementary Excerpt 1. Archived versions of all primary source materials (e.g. ODPRs) are available in a Zenodo archive (3).


```
{
  "id": "CAR1264",
  "opr_id": "CAR1264",
  "arb_id": "CAFR5364",
  "name": "Chugach Alaska Forest Carbon Project",
  "apd": "Forest Carbon Partners, LP",
  "opo": "Chugach Alaska Corporation",
  "owners": ["Chugach Alaska Corporation"],
  "developers": ["New Forests", "Chugachmiut", "Steigerwaldt Land Services", "SilviaTerra"],
  "attestor": "Brian Shillinglaw",
  "is_opo": true,
  "coordinates": [-144.6547, 60.348],
  "shape_centroid": [
    [-141.21765950227456, 59.94224471720261],
    [-143.98927800905210, 60.41272655229106],
    [-146.45145990516664, 60.91395289718339],
    [-147.47519696093053, 59.97624544110733],
    [-147.69887679852079, 60.35266171455008]
  ],
  "supersection_ids": [287],
  "acreage": 49996.0,
  "buffer_contribution": 1326423.0,
  "arbocs": {"issuance": 7143740.0, "calculated": 7536491.664, "reported": 7536492.0},
  "carbon": {
    "initial_carbon_stock": {"value": 206.82, "units": "tCO2e acre-1"},
    "common_practice": {"value": 84.9, "units": "tCO2e acre-1"},
    "average_slag_baseline": {"value": 84.9, "units": "tCO2e acre-1"}
  },
  "baseline": {"ifm_1": 5279314.0, "ifm_3": 463522.0, "ifm_7": 16247.0, "ifm_8": 11804.0},
  "rp_1": {
    "start_date": "2017-06-06", "end_date": "2018-06-06",
    "ifm_1": 12886746.0, "ifm_3": 463522.0, "ifm_7": 0.0, "ifm_8": 0.0,
    "secondary_effects": -21799.0, "confidence_deduction": 0.002
  },
  "assessment_areas": [
    {
      "code": 287, "site_class": "all", "acreage": 49996,
      "species": [
        {"code": 98, "basal_area": 86.8, "fraction": 0.409},
        {"code": 263, "basal_area": 61.9, "fraction": 0.292},
        {"code": 264, "basal_area": 49.1, "fraction": 0.231},
```

```
        {"code": 747, "basal_area": 14.4, "fraction": 0.068}
      ]
    }
  ],
  "notes": "",
  "comment": ""
}
```


**Supplementary Excerpt 1**. Sample project record (CAR1264) from the database.

Our final digitized database contains 93 entries, representing all credited IFM offset projects we were able to identify that were credited as of the 2020-09-09 CARB issuance table (4). We identified 19 of those projects as having participated in the CARB Early Action (EA) program phase and subsequently "graduated" into the compliance program. Reporting details about the first reporting period of these projects required examining far less standardized "project design documents" (PDDs, as opposed to OPDRs). In some cases, project details from the EA project, as reported in the PDD, differed from the values reported in the graduated project's OPDR, raising further concerns about data consistency. Given the less standardized project documentation, combined with the fact that many Early Action projects were initiated under a slightly different set of rules than the final 2011 CARB US Forest Projects protocol, we opted to exclude all Early Action projects from our analysis so as to ensure we applied the same data entry and analysis methods to all projects. This decision also ensures that any rule changes between the EA and the compliance program do not influence our results.

Our primary analysis focused on the 74 remaining projects that entered the CARB offset protocol under the finalized rules of one of the 2011, 2014, and 2015 US Forest Projects protocols. Of those 74 projects, 72 projects received "upfront" offset credits due to the project's initial carbon stocks exceeding protocol-determined common practice. Of those 72 projects, 65 projects could be analyzed using the species classification approach described below. For five of the unanalyzed projects, we were unable to identify a list of species in any publicly available documents (projects ACR248, ACR288, CAR1094, CAR1217, and CAR1032). One project (CAR1102) reported species composition for the entire project, as opposed to per assessment area. Under typical circumstances, our method uses the project wide species composition to estimate standing carbon for each assessment area. However, in this case, CAR1102 spans two supersections and one supersection (Northern California Coast) did not have observations for some of the oak forest types present in the project. This missingness results in the inability to estimate standing live carbon for over 25% of the project's basal area. Rather than make assumptions about how species map to various supersection/assessment area combinations, we excluded the project from consideration. Finally, ACR360, a project in Alaska's Copper River Basin falls entirely within the USFS ecosection 133B. However, there are no FIA plots in ecosection 133B, so we did not include the project in our analysis.

**Verification of crediting calculations**

We verified the accuracy of our digitization by replicating actual project crediting calculations directly from project data, using Equation 5.1 from the 2015 CARB US Forest Project protocol (1). We used the September 9, 2020 version of the California Air Resources Board's Credit Issuance Table as the official record of how many ARBOCs were awarded to each IFM project (4). CARB updates its official issuance table on a bimonthly basis (https://ww2.arb.ca.gov/our-work/programs/compliance-offset-program/arb-offset-credit-issuance).

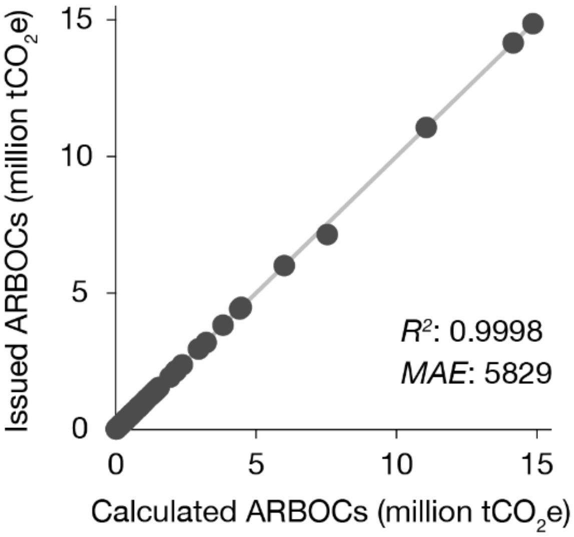


**Supplementary Figure 1**. Comparison between $ARBOC_{Calculated}$, our rederived calculation of ARBOCs awarded to each project from data contained within project OPDRs, and $ARBOC_{Issuance}$, the actual number of ARBOCs awarded to a project by CARB. Mean absolute error of 5829 $tCO_2e$.

Despite obtaining extremely similar results (Supplementary Figure 1), we identified some small differences, which we describe comprehensively in Appendix 1. For clarity, we introduce three pieces of notation to distinguish various offset credit (ARBOC) estimates: $ARBOC_{Issuance}$ refers to ARBOCs issued by CARB and reported in the issuance table, $ARBOC_{Reported}$ refers to ARBOCs reported by offset projects in their OPDRs, and $ARBOC_{Calculated}$ refers to ARBOCs we re-calculated in our analysis by applying our understanding of program rules to the raw data reported in project OPDRs. Two members of our project team (G. Badgley and B. Haya) independently performed this exercise to ensure quality and converged on a unified result. In some instances, we refer to their findings by name. To our knowledge, this work reflects the first public attempt to audit project reported ARBOCs.

**Forest inventory data**

We precomputed estimates of above ground live carbon from the USFS Forest Inventory and Analysis (FIA) database using the rFIA package, an open source software package that implements the queries necessary to replicate USFS statistical procedures (e.g., expansion factors, stratum weighting) for deriving robust inferences from FIA survey data (5).

For every assessment area within every supersection, we calculated above ground live carbon for each forest type code (FORTYPCD) using the `biomass` function from the rFIA software package. Specifically, within rFIA, we loaded data from all US states overlapping the given supersection, matched inventories across states, and removed any samples falling outside the geographic boundary of the supersection. We then calculated above ground live carbon for all accessible, forested conditions (COND_STATUS_CD=1) on private land (OWNGRPCD=40). Finally, CARB common practice estimates for states in the Pacific Northwest work unit (AK, CA, OR, WA) used regional biomass estimates, as opposed to using biomass as reported in the default TREE table (O. Kuegler, personal communication). For these four states, we used regional biomass estimates reported in the TREE_REGIONAL_BIOMASS table. We included these values by setting DRYBIO_SAPLING, DRYBIO_WDLD_SPP, and DRYBIO_TOP equal to zero, retaining DRYBIO_STUMP, and replacing DRYBIO_BOLE with the reported per-tree value of REGIONAL_DRYBIOT from the TREE_REGIONAL_BIOMASS table.

When supersections spanned multiple states we harmonized FIA evaluations across all states using the rFIA function clipFIA, with option matchEval set to TRUE.  We subset data spatially using the `polys` argument in `biomass`, meaning that plots were assigned to as supersection based on the “fuzzed,” publicly reported latitude and longitude values. We used the temporally indifferent method (“TI”), meaning our standing live carbon estimates pool together all FIA survey panels within a single inventory period. Whenever possible, we reported the carbon estimates as the median of inventories ending between 2010 and 2013, so as to be consistent with the snapshot of FIA data used by CARB to produce its own estimates of common practice. In the rare cases where no inventory period ended between 2010 and 2013, we took the median of all inventories from 2013 onward.

These queries yielded a point estimate and variance for above ground carbon and forested area for each forest type code and inventory. These estimates provide the inputs into our subsequent analyses.

**Verification of common practice**

Given an estimate of carbon for each forest type, we can estimate different versions of common practice by aggregating in different ways. To validate our use of FIA data, we first used our carbon estimates to compute common practice as computed by CARB.

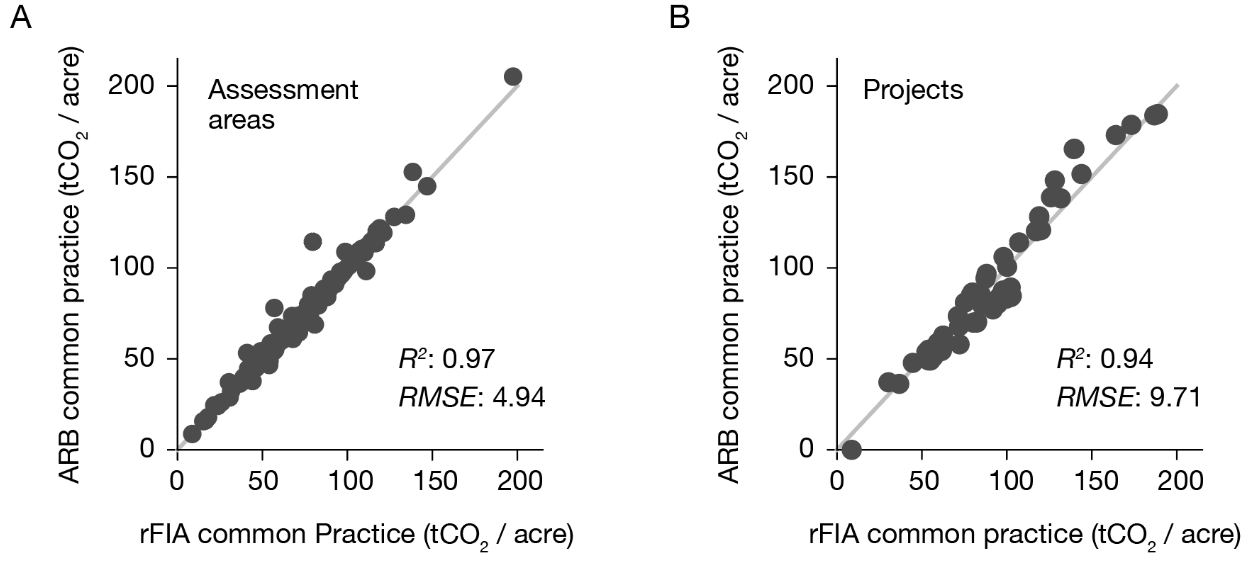


**Supplementary Figure 2**. Comparison of common practice per assessment area (A) and on a per project (B) basis.

First, for each assessment area, we aggregated our carbon estimates within the assessment area, and compared the result directly to the value reported by CARB in 2015. Across all assessment areas containing projects, we found extremely high similarity ($R^2$=0.97, RMSE=4.94 $tCO_2e$/acre). We limited our comparison to supersections containing credited projects.

Second, for each project, we used the project-reported fractional decomposition by assessment area to compute a weighted average for the project ($CP_0$), and compared these to common practice as reported by individual projects in project documentation ($CP_{ARB}$). We found high agreement ($R^2$=0.94, RMSE=9.71). On average, our estimates ($CP_0$) were 3.2% higher than CARB's reported values ($CP_{ARB}$).

Minor deviations in both cases could be due to differences in exact inventories used as well as revisions to the underlying data and stratifications. Because FIA data archives can be updated, the newer version of the FIA database we use might have slightly revised data as compared to the database used by CAR and CARB. In particular, projects were registered under three different versions of CARB's US Forest Project protocol that were issued in 2011, 2014, and

2015. The handling of a concept called site class in the protocol changed in 2015, and our estimates use the approach CARB employs in its 2015 protocol.

As outlined in the Brief methods and described in detail below, these small differences are highly unlikely to influence our analysis of over- or -under- crediting because we calculate *proportional changes* in common practice, each derived from the same underlying data, thereby isolating the effect of how FIA data is aggregated to calculate common practice, as opposed to uninformative differences between our estimates of common practice and the FIA values used by in the CARB US Forest Project protocol.

Together, these results validate our ability to compute common practice from FIA data, and thus allow us to consider variants of common practice calculated using alternate aggregations.

**Classification algorithms**

Our analysis of over- and under-crediting relies on an alternate method of calculating common practice based on the species-specific composition of each project. This calculation relies on a radius-neighbors classifier that maps the species composition (as reported in project OPDRs) to forest types (as reported by FIA on a per-condition basis). Here, we describe that algorithm in detail.

Intuitively, the classifier takes as input the fraction of each species, and produces as output the probabilities of it belonging to one of several forest type codes. We implemented the classifier using the `RadiusNeighborsClassifier` method from scikit-learn (6). Rather than look for n-nearest neighbors, the `RadiusNeighborsClassifier` produces a classification estimate based on all training data that falls within a fixed radius of the observation. This approach is useful when classifying observations within potentially sparse "neighborhoods." To train the classifier, we used pairs of two observed quantities on a per-condition basis — fractional basal area per species and recorded forest type code. We trained a separate classifier for each supersection. Grid search was used to find the radius that maximized performance with 5-fold cross-validation. Supplementary Table 1 reports the weighted F-1 accuracy scores of the final models, as evaluated on a 20% hold-out sample. F-1 scores are the harmonic mean of classifier recall and precision ($\frac{2(precision \cdot recall)}{precision + recall}$) with a score of 0 being the worst score possible and 1 being the best.

After training the classifier, for each assessment area within each project, we used the reported species composition to estimate a forest type code distribution, and used that distribution in our alternate common practice calculation. Although most projects report species composition in terms of a per-species fractional basal area for each assessment area, 24 projects instead report species composition for the entire project. In these cases, we used the classifier as above, assigning the whole-project species composition to all assessment areas.

As a final check, we screened classifier performance by comparing the project's species list against the classifier outputs using the outputs shown in Appendix 2.

| Supersection | Weighted F1-Score |
|---|---|
| Gulf Coastal Plain | 0.74 |
| Northern California Coast | 0.75 |
| Southern Cascades | 0.82 |
| Laurentian Mixed Forest Northern Highlands | 0.77 |
| Adirondacks & Green Mountains | 0.74 |
| St Lawrence & Mohawk Valley | 0.76 |
| Okanogan Highland | 0.88 |
| Columbia Basin | 0.89 |
| White Mountains - San Francisco Peaks - Mongollon | 0.93 |
| Lower New England - Northern Appalachia | 0.73 |
| Central New Mexico | 0.94 |
| Northwest Cascades | 0.87 |
| Allegheny & North Cumberland Mountains | 0.69 |
| Southern Allegheny Plateau | 0.68 |
| Laurentian Mixed Forest Southern Superior | 0.79 |
| Eastern Broadleaf Forest Cumberland Plateau | 0.69 |
| White Mountains | 0.78 |
| Maine - New Brunswick Foothills and Lowlands | 0.78 |
| Southeast and South Central Alaska | 0.91 |

**Supplementary Table 1**. Weighted F1-scores for the per supersection classifiers. F1-scores provide a weighted average of classification recall and precision, with 1 being the highest possible value.

**Calculation of under- and over-crediting**

Our analysis of over- and under-crediting considers three versions of common practice: the common practice reported by each project ($CP_{ARB}$), a calculation of common practice meant to be as comparable as possible to the approach used by CARB used ($CP_0$) by aggregating within assessment areas, and a recalculation of common practice using the species classification method described above ($CP_1$).

To calculate $CP_1$, for each project we use the probabilities returned by the classifier to compute a weighted average of $tCO_2$/acre across forest types. This approach aggregates over only the forest types that match the species composition of the project and are within the geographic bounds of the supersection, as opposed to uniformly aggregating over a discrete list of forest type codes in a predefined assessment area, which may not correspond to the actual species in the project. For example, our approach prevents projects that are primarily Douglas Fir to be classified as Pinyon/Juniper (e.g. CAR1183), and it prevents Douglas Fir projects from being compared to an aggregation of Douglas Fir and Ponderosa Pine (e.g. in the Southern Cascades Mixed Conifer assessment area).

Ideally, we would compare our classification result ($CP_1$) to the actual common practice reported by projects ($CP_{ARB}$). However, several factors make this comparison potentially problematic. First, projects have been developed under two different sets of common practice rules, depending on whether projects were developed before and after the adoption of the 2015 offset protocol. It is especially difficult to recreate how common practice values treated "site class" in their calculations in the earlier 2011 and 2014 protocol versions. Prior to the 2015 revision, the cutoff between "high" and "low" site class varied from supersection to supersection (and perhaps even from assessment area to assessment area), making recreating the earlier common practice values exceedingly difficult.

While it is particularly difficult to identify how calculations in the 2011 and 2014 protocols were performed, we have reason to think they relied at least in part on incomplete data, as evidenced by the fact that there were assessment areas that were assigned a common practice of 0 $tCO_2e$/acre, which is biologically impossible. Second, and related, we know that our analysis does not use the same version of FIA that was used to compute all instances of $CP_{ARB}$, because FIA data are updated and can change over time. Furthermore, we could find no public documentation of the data or code used to calculate common practice prior to the 2015 version of the CARB protocol.

Because of these possible sources of error, we avoid comparing $CP_1$ directly to $CP_{ARB}$. Instead, we focus on the sensitivity of common practice calculations to assumptions about aggregation, which ought to be comparable across projects and across time. We can directly calculate that

sensitivity by comparing our estimate of $CP_1$ to our estimate of $CP_0$, both of which are derived from the same preprocessing of the same underlying FIA data. Calculating sensitivity this way isolates the effect on common practice of changing assumptions about aggregation. Given that our estimates of $CP_0$ are highly similar to $CP_{ARB}$, we can confidently use this sensitivity to infer potential under- and over-crediting.

Having calculated the ratio of $CP_1$ to $CP_0$, we calculate a new common practice for each project by using this ratio to rescale the project's actual common practice.

$$CP_{NEW} = (CP_1 / CP_0) * CP_{ARB}$$

We then recalculate the CARB offset credits (ARBOCs) that would have been awarded to the project on the basis of $CP_{NEW}$. Recall that, under the protocol, awarded upfront credits are based on the difference between the IFM-1 project scenario and IFM-1 baseline scenario. Further, the IFM-1 baseline scenario is constrained to be above common practice, and empirically, nearly all projects present a baseline that is at or only slightly above common practice (89% of projects within 5% of common practice). To calculate potential over- or under-crediting, we assume that new IFM-1 baselines would similarly be set to this new common practice. A caveat is that IFM-1 incorporates both above and below ground carbon, which is calculated in the protocol using per-species allometric equations, whereas $CP_{NEW}$ only considers above ground carbon. To correct for this difference, for each project, we estimated the ratio of above ground to below ground carbon by dividing IFM-1 in the project scenario by the initial above ground carbon stock reported by the project, which typically yields a scale factor slightly greater than 1 (1.23 +/- 0.04 mean/sd).

$$\text{Belowground Scalar} = \text{IFM-1 } [tCO_2] / (\text{Initial carbon stock } [tCO_2/acre] * \text{Acreage } [acres])$$

We multiply $CP_{NEW}$ by project acreage and the scale factor, and set this value as IFM-1 in the baseline scenario. Given a new baseline IFM-1 for a project, we can then recalculate ARBOCs using Equation 5.1 of the 2015 US Forest Projects protocol.

We express under- and over-crediting in units of million $tCO_2e$ and also as a percent of the total number of offset credits issued to the project. We also sum under- or over-crediting across projects and express this sum as a fraction of the total ARBOCs of all projects analyzed. We use $ARBOC_{Calculated}$, as opposed to $ARBOC_{Issued}$, to account for the fact that details provided in the digitized records occasionally differ from the documents used by CARB for issuance.

Note that IFM-3 (standing dead carbon) is ignored in this analysis. For the majority of projects (54%), IFM-3 in the baseline scenario and project scenario are equal, suggesting that this is not a major source of credits. We are thus not estimating any over- or under-crediting for IFM-3.

Note also that any systematic bias in our estimates of $CP_0$ relative to $CP_{ARB}$ could potentially overestimate (or underestimate) our re-crediting calculations. Specifically, if we systematically overestimated $CP_0$, then we underestimated over-crediting; similarly, if we systematically underestimated $CP_0$, then we overestimated over-crediting. As reported above, our estimates of $CP_0$ are well matched to $CP_{ARB}$ ($R^2$=0.94, RMSE=9.76), and on average were 3.2% higher than $CP_{ARB}$. If anything, the fact that we overestimate $CP_{ARB}$ likely makes our overall finding of net over-crediting conservative. In addition, we found no evidence for a systematic relationship between error in our estimate of $CP_0$ and our estimates of crediting error (r=0.06).

We used Monte Carlo error propagation to bound our estimates of crediting error. Using variances of total carbon per acre as reported by rFIA, and assuming gaussian noise, we sampled 1,000 random draws of FIA carbon estimates for $CP_1$ and on each draw calculated crediting for individual projects and across the full portfolio of projects. We use these distributions to report 5th, 25th, 50th, 75th, and 95th percentiles for our estimates of crediting error.

In general, variability in our estimates of crediting error was largest when the number of FIA conditions available for analysis was small.

**Special methods for CAR1183**

In one unusual case, CAR1183, we had to slightly modify our primary method due to a factual error in CARB's 2011 and 2014 forest offset protocols. Because we had to change our method for this project, we performed an additional and complementary analysis to evaluate the robustness of our results.

When CAR1183 was initially listed, the entire project was assigned to the Central New Mexico Pinyon/Juniper assessment area, which CARB assigned a common practice ($CP_{ARB}$) of 0 $tCO_2e$ per acre — a clear error, as this number implies forests in the region contain no carbon. Weeks after the project was listed, CARB's 2015 US Forest Projects protocol fixed this error by (i) updating the Central New Mexico Pinyon/Juniper assessment area to have a non-zero common practice and (ii) introducing a "Mixed Conifer" assessment area to the supersection to capture additional forest types that are present in this region.

Despite these revisions, the fact that the project's reported $CP_{ARB}$ (see Extended Methods Equation 1) equaled zero means that our estimate of $CP_{NEW}$ would always equal zero.
This because our method multiplies the ratio of rFIA derived common practice estimates ($CP_1/CP_0$) by $CP_{ARB}$. To avoid this problem, we directly used $CP_1$ to calculate the crediting error

for CAR1183. Using this method, we estimated that 100% of the project's upfront credits were over-credited.

In light of this methodological nuance and the unusual situation of the addition of a new assessment area, we performed a complementary analysis to assess the robustness of our 100% over-crediting result. Instead of asking what would happen if the project baseline had been subjected to our alternative common practice estimate, we asked instead whether the project would have earned any upfront credits under the terms of the 2015 US Forest Projects protocol, which would have applied if the project's paperwork had been filed with CARB a few weeks later.

From the project documentation, we know that 14.1% of the project's basal area is made up of Pinyon/Juniper species. Under the 2015 protocol, the Central New Mexico Pinyon/Juniper assessment area has a common practice of 8.74 $tCO_2e$ per acre and the Central New Mexico "Mixed Conifer" assessment area has a common practice of 42.77 $tCO_2e$ per acre. While we do not know the true acreage that should be classified as Pinyon/Juniper, if we assume that 14.1% of the project acreage would have been assigned to the Pinyon/Juniper assessment area and the remainder to the Mixed Conifer assessment area, the project's realized common practice would be 37.97 $tCO_2e$ per acre [8.74 * 0.141 + 42.77 * (1-0.141)].

Because the project's initial carbon stocks were 35.61 $tCO_2e$ per acre, which is less than 37.97 $tCO_2e$ per acre, our separate calculation indicates that the project would not have been eligible for upfront carbon credits under the terms of the 2015 US Forest Offsets protocol. This result is independent of, and thus complementary to, our primary reclassification-based analysis, which produces the same result.

For our alternate analysis, it is important to note that the CARB protocol requires that project acreage be assigned to assessment areas on an 'area-weighted', as opposed to 'basal-area weighted' basis. However, because the project came in under the 2014 protocol rules, when only a single assessment area (Pinyon/Juniper) existed, no such area-weighted breakdown is provided in the project's documentation. Instead, we make the assumption that species-level basal area serves as a reasonable proxy for project area. This assumption has biases that cut in both directions. On the one hand, basal area could under-predict the project area that is Pinyon/Juniper woodland because these Pinyon/Juniper crowns can be relatively well-spaced and therefore take up a greater share of land to produce a given share of basal area. On the other hand, we know that the Pinyon and Juniper species listed on the initial OPDR co-associate with the Mixed Conifer forest type strata, specifically Ponderosa pine, so less than 100% of the basal area of Pinyon and Juniper species would classified as Pinyon/Juniper woodland. Thus, in the absence of additional information, we believe using basal area as a proxy for total acreage is a reasonable assumption. In order for initial carbon stocks to exceed

the project's common practice number, which is required to award any "upfront" credits to the project, Pinyon/Juniper would need to account for 20% of the total project acreage.

## Spatial arbitrage patterns

For purposes of understanding the finer spatial variations in carbon stocks, we also worked with FIA data on a "per condition" basis. The approach described here was used to create the arbitrage potential map in Figure 6A, but not used elsewhere in our analysis.

For each supersection, we began by loading all FIA conditions for that supersection, as well as all bordering supersections. We then filtered the FIA data to meet the following criteria: (i) classified as accessible forestland (COND_STATUS_CD == 1); (ii) that were measured between 2001 and 2015; and (iii) fell on privately owned land. Using publicly reported (i.e., fuzzed and swapped) plot latitude and longitude, we assigned each condition a mean temperature and mean precipitation based on 30-year climate normals from PRISM (7). PRISM data were first regridded to a 4km Albers Equal Area Projection using area weighted resampling. Though reported FIA coordinates are approximate, the uncertainty in plot location (within ~500 acres) is comparable to the $4km^2$ spatial resolution of the regridded PRISM data. To account for the difference in magnitude between precipitation and mean annual temperature, we transformed both quantities using a quantile transformer, which maps the cumulative distribution function of observed data to a uniform distribution. Each value is mapped (via its quantile) to the new distribution. This approach aids in the comparison of values measured on different scales (here, millimeters and degrees Celsius). Intuitively, a 10 mm change in precipitation is much less drastic than a 10 °C change in temperature. A 10 mm change in precipitation would hardly affect the reported quantile of an observation, whereas a difference of 10 °C would cause a large change in the reported quantile.  Quantizing both measurements facilitates the subsequent analysis of identifying FIA plots in analogous "climate space." Then, for each point in the 4km PRISM climate grid, we looked up the nearest *n* points in climate space, where *n* was set equal to 10% of all conditions (of any forest type) found in (i) the supersection and (ii) its bordering supersections. We then calculated mean standing live above ground carbon across those *n* conditions, taking into account per tree expansions factors (TPA_UNADJ) and condition proportion (CONDPROP_UNADJ). In addition to mean standing live aboveground carbon, we also calculated "relative standing aboveground carbon" by dividing each 4km estimate of mean carbon by the mean of all FIA plots falling within the supersection (e.g., excluding conditions in bordering supersections).

We reiterate that none of our analysis of crediting errors (i.e., Figure 5) uses FIA data on a "per condition" basis in the manner described above. Rather, our primary analysis strictly follows the sampling and stratification rules of FIA survey design per the open-source rFIA package methods. "Per condition" data are only used for demonstrative purposes in Figure 6A to highlight the distinct biogeography of carbon stocks in the Southern Cascades supersection.

## Open source software and data

We performed all analyses using Python and, in the case of rFIA (5), the R programming language (8) in the Pangeo cloud environment (9). Our workflow used the following open source software packages: Pandas (10); Xarray (11); Matplotlib (12); NumPy (13); Seaborn (14); Jupyter (15); Scikit-learn (6). The source code to reproduce our analysis is available in (16, 17). Archival versions of this project's data products are available in (3, 18).

## References


1. California Air Resources Board, Compliance Offset Protocol U.S. Forest Projects (2015).
2. Burrill, Elizabeth A., *et al.*, The Forest Inventory and Analysis Database: Database Description and User Guide for Phase 2 (version 8.0) (2018).
3. G. Badgley, J. Freeman, J. J. Hamman, B. Haya, D. Cullenward, California improved forest management offset project database (Version 1.0.0) https:/doi.org/10.5281/zenodo.4630684.
4. California Air Resources Board, "ARB Offset Credit Issuance Table" (2020).
5. H. Stanke, A. O. Finley, A. S. Weed, B. F. Walters, G. M. Domke, rFIA: An R package for estimation of forest attributes with the US Forest Inventory and Analysis database. *Environmental Modelling & Software* **127**, 104664 (2020).
6. F. Pedregosa, *et al.*, Scikit-learn: Machine Learning in Python. *Journal of Machine Learning Research* **12**, 2825–2830 (2011).
7. PRISM Climate Group, Oregon State University, PRISM 30-year Climate Normals (2016).
8. R. Ihaka, R. Gentleman, R: A Language for Data Analysis and Graphics. *Journal of Computational and Graphical Statistics* **5**, 299–314 (1996).
9. N. H. Robinson, J. Hamman, R. Abernathey, Seven Principles for Effective Scientific Big-DataSystems. *arXiv:1908.03356 [cs]* (2020) (March 3, 2021).
10. W. McKinney, Data Structures for Statistical Computing in Python in (2010), pp. 56–61.
11. S. Hoyer, J. J. Hamman, xarray: N-D labeled Arrays and Datasets in Python. *Journal of Open Research Software* **5**, 10 (2017).
12. J. D. Hunter, Matplotlib: A 2D Graphics Environment. *Comput. Sci. Eng.* **9**, 90–95 (2007).
13. C. R. Harris, *et al.*, Array programming with NumPy. *Nature* **585**, 357–362 (2020).
14. M. Waskom, *et al.*, mwaskom/seaborn (v0.11.1) (2020) https://doi.org/10.5281/zenodo.4379347.
15. T. Kluyver, *et al.*, "Jupyter Notebooks—a publishing format for reproducible computational workflows" in *Positioning and Power in Academic Publishing: Players, Agents and Agendas*, F. Loizides, B. Schmidt, Eds. (IOS Publishing, 2016), pp. 87–90.
16. G. Badgley, *et al.*, carbonplan / forest-offsets (Version 1.1.0) https://doi.org/10.5281/zenodo.5567957.
17. G. Badgley, *et al.*, carbonplan / forest-offsets-paper (Version 1.1.0) https://doi.org/10.5281/zenodo.5567962.
18. G. Badgley, *et al.*, Systematic over-crediting in California's forest carbon offsets program (Version 1.1.0) https://doi.org/10.5281/zenodo.5565457.

## Glossary

| | |
|---|---|
| **Additionality** | When used to describe an offset project, the claim that a project's climate benefits were induced by the offset credit, i.e. that project scenario climate benefits are "in addition to" what would have happened in the baseline scenario or that the climate benefits would not exist without the offset project's activities.<br><br>When used to describe an offsets program, the claim that the climate benefits achieved by the program are equal to or greater than the number of credits awarded to participating projects.<br><br>Additionality is a critical requirement for compliance offsets in particular because offsets that are used in compliance contexts allow higher emissions that are premised on offsets' additional climate benefits. |
| **Air Resources Board Offset Credits (ARBOCs)** | The name for the offset credits issued by the California Air Resources Board. ARBOCs are eligible for compliance use in the state's cap-and-trade program. Each ARBOC is worth 1 metric $tCO_2e$. |
| **Assessment Area** | A forest type that spans the full geographic extent of a supersection. Each supersection contains one or more assessment areas, each with a distinct estimate of common practice that is based on the average carbon stock for this forest type from USFS FIA data. |
| **Basal area** | The cross-sectional area of a tree at breast height. Often used to describe the total cross-sectional area of all trees on a plot. |
| **Baseline scenario** | A carbon offset has a project scenario and a baseline scenario. The baseline scenario describes the emissions outcomes that would happen counterfactually in the absence of an offset project, i.e. what would happen if the offset project is not pursued. By definition, an offset project's baseline scenario cannot be observed because it does not occur. |
| **California Air Resources Board (CARB)** | California's climate change regulator, which is responsible for the state's cap-and-trade and compliance carbon offsets program. CARB imported the core architecture of the US Forest Project protocol from an earlier Climate Action Reserve protocol in its 2011 and 2014 US Forest Projects protocols, then subsequently updated common practice numbers in coordination with USFS for its 2015 protocol revision. |
| **Climate Action Reserve (CAR)** | A nonprofit organization and carbon offset registry that developed the original forest offset program subsequently adopted and then revised by CARB. CAR created supersections and assessment areas, and developed the original common practice numbers adopted without change by the 2011 and 2014 CARB US Forest Projects protocols. |
| **Common practice** | The average carbon stock ($tCO_2$/acre) in a given assessment area, as calculated across an entire supersection. |

| | |
|---|---|
| **Compliance offsets** | Carbon offsets that are fungible for compliance purposes in legally binding climate mitigation policies. Here, California has a cap-and-trade program that sets aggregate limits on greenhouse gas pollution. Because forest carbon offsets can be used by polluters to comply with the cap-and-trade program, forest carbon offsets are known as compliance offsets. Compliance offsets increase the total emissions allowed under climate mitigation policies premised on their claim to generate equivalent climate benefits elsewhere. |
| **$CP_{ARB}$** | Common practice as reported by individual projects in the CARB program, expressed in $tCO_2$/acre as a weighted average of all project assessment areas. |
| **$CP_0$** | This study's re-calculation of common practice directly from FIA data, matching methods used by CARB and expressed in $tCO_2$/acre. |
| **$CP_1$** | This study's re-calculation of common practice using a classification model to match projects' actual species with comparable FIA plots in order to minimize ecological bias, expressed in $tCO_2$/acre. |
| **Ecosection** | A geographic region defined by the USFS. CAR combined multiple individual ecosections together to form supersections, which serve as the geographic aggregations across which assessment areas are defined and common practice is calculated. |
| **Initial carbon stock (ICS)** | A measure of the standing live aboveground carbon stock in a given forest. Improved forest management (IFM) projects receive large upfront credits in their first reporting period (RP1) when their ICS exceeds the 100-year average carbon stock in their baseline scenario. |
| **Improved Forest Management (IFM)** | A kind of forest offset project that claims to increase average carbon stocks over time by changing the rotation or other management techniques affecting forest growth and harvest cycles. In California's US Forest Projects protocol, IFM credits receive "upfront" credit based on the difference between their initial standing carbon stock (as measured by site surveys) and the 100-year average of carbon stocks in their projected counterfactual baseline scenario. For IFM projects that have initial standing carbon stock above common practice, the 100-year average of carbon stocks in their baseline scenario must be equal to or greater to common practice. |
| **IFM-1** | A component of the "GHG boundary" for which greenhouse gas emission reductions can be credited, representing onsite standing live tree carbon, both above and belowground, in either the baseline or project scenario. Reported in an offset project's OPDR. |
| **IFM-3** | A component of the "GHG boundary" for which greenhouse gas emission reductions can be credited, representing the amount of onsite standing dead tree carbon, in either the baseline or project scenario. Reported in an offset project's OPDR. |

| | |
|---|---|
| **IFM-7** | A component of the “GHG boundary” for which greenhouse gas emission reductions can be credited, representing the amount of carbon stored within “in-use” wood products, in either the baseline or project scenario. Reported in an offset project’s OPDR. |
| **IFM-8** | A component of the “GHG boundary” for which greenhouse gas emission reductions can be credited, representing the amount of carbon stored within “land-filled” wood products, in either the baseline or project scenario. Reported in an offset project’s OPDR. |
| **Forest Inventory and Analysis (FIA)** | A comprehensive dataset describing forests and forest ecology in the United States, collected and maintained by the US Forest Service. |
| **Non-additional** | When a project or program fails to achieve the additionality standard. |
| **Offset credit** | In the context of California’s program, an offset credit represents 1 $tCO_2e$ using 100-year IPCC AR4 global warming potentials. In California’s program, offset credits are called Air Resources Board Offset Credits (ARBOCs). |
| **Offset project data reports (OPDRs)** | A standardized public reporting document required for each offset project and filed with one of the private carbon offset registries that helps CARB implement its compliance offset program. OPDRs contain critical information about offset projects, including species-level data, baseline, and project scenario information, and serve as the basis for the calculations a project developer makes to claim credits from CARB. Using a critical subset of these data (IFM-1, IFM-3, IFM-7, and IFM-8) we are able to re-calculate the number of offset credits that should be issued to any publicly listed offset project with an OPDR. |
| **Over-crediting** | The outcome in which a project is awarded more credits than the climate benefits it can rightly claim. In this study, we report over-crediting when our re-estimate of common practice leads to a higher number than what a project uses to earn credits under the US Forest Projects protocol. |
| **Project scenario** | A carbon offset has a project scenario and a baseline scenario. The project scenario is the scenario that describes the emissions outcomes when an offset project is implemented, i.e. what a project claims will happen in reality if pursued. |
| **RP1** | Reporting Period 1, the first reporting period for offset projects’ documentation. All upfront credits are awarded in RP1, along with the first tranche of annual credits that reflect forest growth. |
| **RP2** | Reporting Period 2, the second reporting period for offsets’ project documentation. The second tranche of annual credits that reflect forest growth are awarded in RP2. Used here to help verify crediting calculations. |
| **Standardized approach to carbon offsets** | A paradigm for offset program regulation. Earlier offset programs used project-level calculations and bespoke methods that regulators approved |

| | |
|---|---|
| | on a case-by-case basis. Standardized offset programs have common rules that establish what kinds of projects are eligible, how project baselines scenarios are determined, and how to calculate the number of offset credits an eligible project should earn. |
| **Supersection** | The geographic unit of analysis in California's forest offsets protocol. CAR originally created supersections in the continental United States by combining together multiple ecosections to form supersections. Each supersection has one or more assessment areas, each with a distinct estimate of common practice. CARB adopted CAR's supersections for use in its compliance offsets program, adding Alaska in its 2015 US Forest Projects protocol. |
| **US Forest Projects protocol** | The California Air Resources Board's forest offset protocol, which sets out standardized rules for project eligibility, baseline, and crediting calculations. CARB has adopted three versions of the protocol: the first in 2011, the second in 2014, and the third and current version in 2015. This protocol produces over 80% of the offset credits in California's cap-and-trade program. All versions have been open to projects anywhere in the continental United States; as of the 2015 version, projects in southern coastal Alaska became eligible as well. |
| **US Forest Service (USFS)** | A branch of the US Department of Agriculture that is responsible for collecting and maintaining the FIA data used here (among other matters). |
| **Under-crediting** | The outcome in which a project is awarded fewer credits than the climate benefits it can rightly claim. In this study, we report under-crediting when our re-estimate of common practice leads to a lower number than what a project uses to earn credits under the US Forest Projects protocol. |
| **Upfront credits** | The credits received by an improved forest management (IFM) project in its first reporting period (RP1). In RP1, most OPDRs do not distinguish between (1) annual forest growth and (2) the much larger number of credits awarded to IFM projects with initial carbon stocks above 100-year average carbon stocks in projects' baselines, so we report both components as upfront credits. |
| **Voluntary offsets** | Carbon offsets that are bought and sold for voluntary, typically private purposes, such as an individual or company wanting to claim carbon neutrality. California's offsets program is a compliance offsets program, not a voluntary program. |

**Appendix 1: Verification of crediting calculations**

Here, we describe a project-by-project description of projects where we identified a discrepancy between the number of credits a project's documentation claims in its official reporting ($ARBOC_{Reported}$), the number of credits th California Air Resources Board issues to the project ($ARBOC_{Issuance}$), and our independent effort to recalculate the appropriate number of credits from projects documentation ($ARBOC_{Calculated}$).

The discrepancies can be categorized in two groups. The first group includes projects for which the number of offset credits reported by offset projects is not equal to what the regulator issued ($ARBOC_{Reported} \neq ARBOC_{Issuance}$), and the second group includes projects for which the number of offset credits reported by offset projects is not equal to what our independent calculations ($ARBOC_{Reported} \neq ARBOC_{Calculated}$). We address each in turn.

<u>Reported not equal to issuance</u>

We start with cases where $ARBOC_{Reported}$ is not equal to $ARBOC_{Issuance}$, further subdividing these cases into four sub-groupings. For each sub-grouping, we list each instance of a discrepancy.

*Unexplained — potential over-crediting*

| | |
|---|---|
| **CAR1175** | Difference of 30 ARBOCs between Reported and Issuance. RP1 has two verification statements — one for 3,824,257 and another for 3,824,227. The annual OPDR for RP2 also records 3,824,227 ARBOCs for RP1. It is possible that CARB over-issued 30 ARBOCs. It does not appear 30 ARBOCs were deducted from a later reporting period. Both G. Badgley and B. Haya calculate a 30 ARBOC difference. |

*Unexplained — possible out–of–date documents*

In all the instances listed below, we've tried to triangulate what the project owner/developer formally requested from CARB. It is our impression that these five projects have updated OPDRs that have not been posted to the registries.

| | |
|---|---|
| **CAR1213** | The Initial OPDR has a "Form Completed" date that is more recent than Annual OPDR for Reporting Period one. The newer initial OPDR reports a different baseline than the verified annual OPDR. It appears that the annual OPDR and the verification statement for RP1 is likely out of date. |
| **CAR1215** | v2.4 of the annual OPDR for RP1 disagrees with the issuance table. The verification statement for RP1, however, agrees with the issuance table. It is likely the case that the most up-to-date OPDR has not been posted. |

| | |
|---|---|
| **CAR1257** | Initial OPDR is more recent than annual OPDR for RP1. It seems likely that the most recent annual OPDR has simply not been uploaded. |
| **CAR1264** | The initial OPDR, which doubles as the annual OPDR for RP1, seems to be out of date. The current version uploaded to the CAR registry portal asserts the project has a reversal rating of 17.6%, whereas CARB used a reversal risk of 10.6% for setting aside this project's buffer contribution in RP1. Furthermore, the verification document for RP1 reports that the final OPDR's total GHG deductions amounted to 7,143,740 ARBOCs. This verified amount matches CARB's issuance but differs from the annual OPDR. |
| **VCSOPR10** | The annual OPDR and verification of the annual OPDR for the first reporting period differ from the value listed in the CARB issuance table. No note or additional information provided by the registry. |

*Correctable Errors*

Two discrepancies arise from projects that have "Correctable Error" notes including in the project documents listed at the registry. These notes indicate that the regulator has taken an action to modify the number of credits reported by the project, but without additional explanation.

| | |
|---|---|
| **CAR1103** | On 11/29/2016, CAR issued a Project Note that states that "During [CARB's] regulatory review, [CARB] identified a correctable error." The note specifies that 270,943 ARBOCs were issued, a number that matches the CARB issuance table. We could not identify a copy of the updated/corrected OPDR. |
| **CAR1208** | On 5/14/20, CAR issued a Project Note that states that "During [CARB's] regulatory review, [CARB] identified a correctable error." The note specifies that 501,850 ARBOCs were issued, a number that matches the CARB issuance table. We could not identify a copy of the updated/corrected OPDR. |

<u>Reported not equal to calculated</u>

We now move to cases where $ARBOC_{Reported}$ is not equal to $ARBOC_{Calculated}$, again subdividing discrepancies into relevant sub-groupings.

*Rounding confidence deduction*

Projects report a confidence deduction to adjust for uncertainty estimates of onsite carbon stocks. We identified three projects where the confidence deduction has been rounded, causing differences between $ARBOC_{Reported}$ and $ARBOC_{Calculated}$.

| | |
|---|---|
| **ACR282** | OPDR reports a confidence deduction of 0.3%. We were only able to arrive at the value for $ARBOC_{Reported}$ if we assumed a confidence deduction of 0.00%. It seems likely that an interim step rounded the confidence deduction to zero, resulting in an over-crediting of approximately 9,171 ARBOCs. However, this difference is made up in RP2. |
| **ACR360** | OPDR reports confidence deduction of 0.67%. However, we were only able to recreate $ARBOC_{Reported}$ when we assumed the confidence deduction was approximately equal to 0.66531%. Yields a difference in $ARBOC_{Reported}$ and $ARBOC_{Calculated}$ of 1,893 ARBOCs. |
| **ACR427** | OPDR reports a confidence deduction of 2.445%.We were only able to recreate $ARBOC_{Reported}$ when we assumed the confidence deduction was equal to 2.4%. Depending on how rounding is treated, could be over-crediting. Yields a difference in $ARBOC_{Reported}$ and $ARBOC_{Calculated}$ of 4,096 ARBOCs. |

*Harvest*

The Forest Offset Protocol has special provisions for landfilled wood products in reporting periods where the cumulative sum of on-site harvested carbon exceeds the cumulative sum of harvested carbon in the baseline scenario. When this condition is satisfied (as outlined by Equation C.2 of the 2011 and 2014 protocols and Equation C.8 of the 2015 protocol), landfilled wood products (IFM-8) are ineligible for crediting. Based on inspection of initial OPDRs, we identified three projects where this extra provision of the protocol was necessary to re-calculate issuance numbers: ACR247, ACR276, and CAR1217. When calculating the number of ARBOCs issued to these three projects, we explicitly set IFM-8 in both the baseline and project scenario to zero. After implementing this logic, $ARBOC_{Calculated}$ nearly matched $ARBOC_{Reported}$ in all cases.

| | |
|---|---|
| **ACR247** | $ARBOC_{Calculated}$ greater than $ARBOC_{Reported}$ by 23. |
| **CAR1217** | $ARBOC_{Calculated}$ equals $ARBOC_{Reported}$ |

| | |
|---|---|
| **ACR276** | $ARBOC_{Calculated}$ less than $ARBOC_{Reported}$ by 0.8 (possible rounding issue). |

*Errors under 25 ARBOCs that might explained by confidence deduction rounding*

All these projects have smaller differences in $ARBOC_{Reported}$ as compared to $ARBOC_{Calculated}$. However, all these projects also have a confidence deduction greater than 0. Therefore, we cannot rule out that rounding of the confidence deduction is the source of the difference. Differences reported below have been rounded.

| | |
|---|---|
| **CAR1094** | Off by 3. |
| **CAR1204** | Off by 14. |
| **ACR256** | Off by 21. |
| **ACR257** | Off by 2. |

*Errors under 25 ARBOCs that cannot be explained by confidence deduction rounding*

This project is off by precisely two. Likely a data entry issue somewhere in the OPDR.

| | |
|---|---|
| **CAR1032** | Off by 2; whole value so likely not rounding. |

*De minimis errors (< 2 ARBOC) that can be explained by leakage/CD rounding*

All these projects have *even smaller* differences in $ARBOC_{Reported}$ as compared to $ARBOC_{Calculated}$. However, all these projects also have a confidence deduction greater than 0. Therefore, we cannot rule out rounding of the confidence dedication as the source of the difference.

| | |
|---|---|
| **ACR260** | No additional comment. |
| **ACR288** | No additional comment. |
| **CAR1314** | No additional comment. |
| **ACR423** | No additional comment. |

| | |
|---|---|
| **ACR182** | No additional comment. |

*Errors of less than or equal to two, not explained by confidence deduction*

These are projects where the confidence deduction of the first reporting period is zero. That means rounding cannot fully explain the difference. It's still possible that intermediate rounding of leakage on wood products could partially explain these differences.

| | |
|---|---|
| **CAR1066** | No additional comment. |
| **ACR393** | Off by exactly 1. |

## Appendix 2: Classification labels

Here we report species composition data and output of our classifier (described above in "Classification algorithm") for all of the 65 projects included in the crediting error analysis reported in Figure 5. For brevity, we exclude listings in the "Project species" and "Forest type classification" columns that fall below 10% from this table; however, all digitized listings are used in the underlying analysis and available as part of our public data.

| Project | Supersection | Assessment Area | Project species (fractional basal area) | Forest type classification probabilities |
|---|---|---|---|---|
| CAR1205 | 2 | 999 | Chestnut oak : 15.5%<br>Yellow-poplar : 14.0% | Yellow-poplar / white oak / northern red oak : 19.8%<br>White oak / red oak / hickory : 34.8%<br>Chestnut oak / black oak / scarlet oak : 38.1% |
| CAR1205 | 76 | 999 | Chestnut oak : 15.5%<br>Yellow-poplar : 14.0% | Yellow-poplar / white oak / northern red oak : 18.8%<br>Chestnut oak / black oak / scarlet oak : 34.8%<br>White oak / red oak / hickory : 39.7% |
| VCSOPR10 | 15 | 999 | Black walnut : 15.2%<br>Shortleaf pine : 15.6%<br>White oak : 29.2% | Shortleaf pine / oak : 12.5%<br>White oak : 16.9%<br>White oak / red oak / hickory : 68.2% |
| ACR192 | 4 | 999 | Longleaf pine : 32.9%<br>Loblolly pine : 19.3%<br>Laurel oak : 18.6% | Longleaf pine / oak : 15.1%<br>Loblolly pine : 15.8%<br>Longleaf pine : 63.7% |
| ACR247 | 1 | 1 | Balsam fir : 38.2%<br>Red maple : 17.6%<br>Yellow birch : 14.3% | Sugar maple / beech / yellow birch : 83.0% |
| ACR247 | 1 | 2 | Red maple : 17.7%<br>Sugar maple : 30.8%<br>Yellow birch : 13.8%<br>Beech : 14.5% | Sugar maple / beech / yellow birch : 99.0% |
| ACR262 | 55 | 221 | Douglas fir : 35.0%<br>Tanoak : 29.0% | Douglas fir : 22.9%<br>Tanoak : 64.6% |
| ACR262 | 79 | 297 | Douglas fir : 35.0% | Douglas fir : 18.1% |

| | | | | |
|---|---|---|---|---|
| | | | Tanoak : 29.0% | Tanoak : 72.8% |
| ACR257 | 32 | 113 | Loblolly pine : 64.8% | Loblolly pine : 92.2% |
| ACR280 | 2 | 999 | Chestnut oak : 11.5%<br>Red maple : 10.8%<br>Yellow-poplar : 14.5% | Chestnut oak / black oak / scarlet oak : 25.4%<br>White oak / red oak / hickory : 28.7%<br>Yellow-poplar / white oak / northern red oak : 31.4% |
| CAR1190 | 55 | 221 | Tanoak : 23.0%<br>Redwood : 49.0%<br>Douglas fir : 24.0% | Tanoak : 26.6%<br>Douglas fir : 28.5%<br>Redwood : 39.7% |
| CAR1208 | 2 | 3 | Yellow-poplar : 27.2%<br>Eastern hemlock : 24.4%<br>Virginia pine : 17.1% | Virginia pine / southern red oak : 13.5%<br>Yellow-poplar / white oak / northern red oak : 52.5% |
| CAR1208 | 2 | 4 | Yellow-poplar : 21.6%<br>Sycamore : 10.6% | Cherry / white ash / yellow-poplar : 10.4%<br>White oak / red oak / hickory : 20.8%<br>Yellow-poplar / white oak / northern red oak : 46.5% |
| CAR1208 | 2 | 7 | Sugar maple : 17.6%<br>Yellow-poplar : 14.4% | Sugar maple / beech / yellow birch : 11.3%<br>Yellow-poplar / white oak / northern red oak : 33.0%<br>White oak / red oak / hickory : 35.5% |
| CAR1208 | 2 | 8 | Chestnut oak : 14.8%<br>White oak : 10.9%<br>Black oak : 10.6% | White oak / red oak / hickory : 42.5%<br>Chestnut oak / black oak / scarlet oak : 47.5% |
| CAR1208 | 2 | 5 | White oak : 24.2%<br>Beech : 22.4%<br>Yellow-poplar : 12.3%<br>Red maple : 11.0% | Yellow-poplar / white oak / northern red oak : 35.4%<br>White oak / red oak / hickory : 51.0% |
| CAR1208 | 24 | 86 | Yellow-poplar : 27.2%<br>Eastern hemlock : 24.4%<br>Virginia pine : 17.1% | Yellow-poplar / white oak / northern red oak : 58.4% |

| | | | | |
|---|---|---|---|---|
| CAR1208 | 24 | 87 | Yellow-poplar : 21.6%<br>Sycamore : 10.6% | White oak / red oak / hickory : 23.1%<br>Yellow-poplar / white oak / northern red oak : 43.1% |
| CAR1208 | 24 | 89 | Sugar maple : 17.6%<br>Yellow-poplar : 14.4% | Yellow-poplar / white oak / northern red oak : 32.9%<br>White oak / red oak / hickory : 40.0% |
| CAR1208 | 24 | 90 | Chestnut oak : 14.8%<br>White oak : 10.9%<br>Black oak : 10.6% | White oak / red oak / hickory : 44.7%<br>Chestnut oak / black oak / scarlet oak : 46.0% |
| CAR1208 | 24 | 92 | White oak : 24.2%<br>Beech : 22.4%<br>Yellow-poplar : 12.3%<br>Red maple : 11.0% | Yellow-poplar / white oak / northern red oak : 30.0%<br>White oak / red oak / hickory : 57.6% |
| CAR1264 | 287 | 287 | Sitka spruce : 40.9%<br>Western hemlock : 29.2%<br>Mountain hemlock : 23.1% | Mountain hemlock : 19.5%<br>Sitka spruce : 34.3%<br>Western hemlock : 46.1% |
| ACR393 | 39 | 151 | Northern white-cedar : 54.0% | Northern white-cedar : 85.9% |
| ACR393 | 39 | 152 | Eastern hemlock : 15.0%<br>Red maple : 21.0%<br>Sugar maple : 31.0%<br>Yellow birch : 11.0% | Sugar maple / beech / yellow birch : 99.2% |
| ACR393 | 39 | 153 | Balsam fir : 12.0%<br>Eastern hemlock : 18.0%<br>Eastern whitepine : 12.0%<br>Northern white-cedar : 20.0%<br>Red maple : 23.0% | Northern white-cedar : 15.1%<br>Sugar maple / beech / yellow birch : 48.4% |
| ACR423 | 58 | 229 | Douglas fir : 71.0%<br>Western hemlock : 13.0% | Douglas fir : 95.2% |
| CAR1314 | 60 | 231 | Ponderosa pine : 57.0% | Ponderosa pine : 43.9% |

| | | | | |
|---|---|---|---|---|
| | | | Douglas fir : 57.0% | Douglas fir : 55.6% |
| CAR1314 | 60 | 232 | Ponderosa pine : 66.0%<br>Douglas fir : 30.0% | Douglas fir : 11.8%<br>Ponderosa pine : 87.3% |
| CAR1314 | 22 | 82 | Ponderosa pine : 63.0% | Ponderosa pine : 94.1% |
| ACR425 | 286 | 286 | Western hemlock : 79.0%<br>White spruce : 14.0% | Western hemlock : 99.0% |
| ACR427 | 94 | 347 | Balsam fir : 17.2%<br>Black spruce : 16.0%<br>Red spruce : 11.5%<br>Northern white-cedar : 20.0% | Black spruce : 10.0%<br>Balsam fir : 71.0% |
| ACR458 | 286 | 286 | Western hemlock : 45.0%<br>Western red cedar : 30.0%<br>Sitka spruce : 11.0% | Western redcedar : 27.8%<br>Western hemlock : 72.2% |
| CAR1180 | 55 | 221 | Douglas fir : 34.0%<br>Redwood : 13.0%<br>Tanoak : 24.0% | Douglas fir : 33.0%<br>Tanoak : 55.5% |
| ACR249 | 2 | 999 | Chestnut oak : 12.2%<br>Yellow-poplar : 16.0% | White oak / red oak / hickory : 26.3%<br>Chestnut oak / black oak / scarlet oak : 27.9%<br>Yellow-poplar / white oak / northern red oak : 36.7% |
| CAR1204 | 42 | 169 | Red maple : 31.4%<br>Sugar maple : 10.5% | Sugar maple / beech / yellow birch : 98.3% |
| CAR1204 | 42 | 171 | Aspen : 32.3%<br>Red maple : 13.2%<br>Balsam fir : 11.7%<br>Red spruce : 10.1% | Sugar maple / beech / yellow birch : 30.2%<br>Aspen : 69.8% |
| CAR1204 | 42 | 172 | Red spruce : 24.8%<br>Northern white-cedar : 19.1%<br>Red maple : 15.1%<br>Balsam fir : 14.2% | Sugar maple / beech / yellow birch : 10.9%<br>Balsam fir : 10.9%<br>Red spruce / balsam fir : 22.9%<br>Red spruce : 49.0% |

| | | | | |
|---|---|---|---|---|
| CAR1204 | 94 | 347 | Red spruce : 24.8%<br>Northern white-cedar : 19.1%<br>Red maple : 15.1%<br>Balsam fir : 14.2% | Balsam fir : 11.0%<br>Sugar maple / beech / yellow birch : 11.8%<br>Red spruce / balsam fir : 21.2%<br>Red spruce : 49.5% |
| CAR1204 | 94 | 348 | Red maple : 31.4%<br>Sugar maple : 10.5% | Sugar maple / beech / yellow birch : 97.8% |
| ACR200 | 55 | 221 | Tanoak : 36.0%<br>Douglas fir : 32.0%<br>Redwood : 21.0% | Douglas fir : 21.6%<br>Tanoak : 75.4% |
| ACR256 | 24 | 999 | Chestnut oak : 29.1% | White oak / red oak / hickory : 17.4%<br>Chestnut oak : 20.7%<br>Chestnut oak / black oak / scarlet oak : 56.2% |
| ACR256 | 69 | 999 | Chestnut oak : 29.1% | White oak / red oak / hickory : 20.3%<br>Chestnut oak : 23.7%<br>Chestnut oak / black oak / scarlet oak : 46.5% |
| CAR1013 | 55 | 999 | Tanoak : 30.9%<br>Douglas fir : 27.8%<br>Redwood : 16.7% | Douglas fir : 20.3%<br>Tanoak : 74.6% |
| CAR1213 | 1 | 1 | Red spruce : 40.9%<br>Balsam fir : 16.3%<br>Blasck cherry : 11.2% | Sugar maple / beech / yellow birch : 10.8%<br>Red spruce : 14.3%<br>Red spruce / balsam fir : 68.3% |
| CAR1213 | 1 | 2 | Red maple : 12.2%<br>Beech : 31.4%<br>Sugar maple : 13.3%<br>Yellow birch : 14.9% | Sugar maple / beech / yellow birch : 100.0% |
| CAR1213 | 86 | 318 | Red spruce : 40.9%<br>Balsam fir : 16.3%<br>Blasck cherry : 11.2% | Sugar maple / beech / yellow birch : 11.4%<br>Red spruce : 13.2%<br>Red spruce / balsam fir : 69.6% |
| CAR1213 | 86 | 319 | Red maple : 12.2%<br>Beech : 31.4%<br>Sugar maple : 13.3%<br>Yellow birch : 14.9% | Sugar maple / beech / yellow birch : 100.0% |

| | | | | |
|---|---|---|---|---|
| ACR199 | 94 | 348 | Sugar maple : 39.5%<br>Yellow birch : 25.9% | Sugar maple / beech / yellow birch : 100.0% |
| ACR199 | 94 | 347 | Balsam fir : 38.1%<br>Red spruce : 17.7%<br>Yellow birch : 17.7% | Balsam fir : 12.9%<br>Red spruce / balsam fir : 26.7%<br>Sugar maple / beech / yellow birch : 47.0% |
| ACR182 | 55 | 221 | Douglas fir : 34.2%<br>Redwood : 15.8%<br>Pacific madrone : 11.6%<br>Tanoak : 25.8% | Douglas fir : 27.7%<br>Tanoak : 59.2% |
| ACR279 | 2 | 999 | Chestnut oak : 11.6%<br>Red maple : 19.0%<br>Yellow-poplar : 18.4% | White oak / red oak / hickory : 18.4%<br>Chestnut oak / black oak / scarlet oak : 25.1%<br>Yellow-poplar / white oak / northern red oak : 31.3% |
| ACR267 | 2 | 999 | Chestnut oak : 12.8%<br>Yellow-poplar : 17.7% | White oak / red oak / hickory : 22.7%<br>Chestnut oak / black oak / scarlet oak : 26.5%<br>Yellow-poplar / white oak / northern red oak : 36.4% |
| CAR1183 | 18 | 71 | Douglas fir : 37.1%<br>Ponderosa pine : 22.9%<br>White fir : 12.8% | Ponderosa pine : 28.5%<br>Douglas fir : 68.4% |
| ACR202 | 38 | 999 | Eastern hemlock : 11.9%<br>Northern white-cedar : 16.5%<br>Sugar maple : 34.5% | Hard maple / basswood : 18.1%<br>Sugar maple / beech / yellow birch : 81.9% |
| ACR202 | 35 | 999 | Eastern hemlock : 11.9%<br>Northern white-cedar : 16.5%<br>Sugar maple : 34.5% | Hard maple / basswood : 12.8%<br>Sugar maple / beech / yellow birch : 87.2% |
| ACR361 | 287 | 287 | Sitka spruce : 94.9% | Sitka spruce : 98.0% |

| | | | | |
|---|---|---|---|---|
| CAR1197 | 1 | 1 | Eastern hemlock : 23.2%<br>Red spruce : 20.2%<br>Red maple : 11.1%<br>Balsam fir : 11.0% | Red spruce : 12.0%<br>Eastern hemlock : 18.7%<br>Sugar maple / beech / yellow birch : 58.3% |
| CAR1197 | 1 | 2 | Beech : 33.7%<br>Sugar maple : 28.2%<br>Yellow birch : 10.8% | Sugar maple / beech / yellow birch : 99.8% |
| CAR1197 | 86 | 318 | Eastern hemlock : 23.2%<br>Red spruce : 20.2%<br>Red maple : 11.1%<br>Balsam fir : 11.0% | Red spruce : 15.0%<br>Eastern hemlock : 21.9%<br>Sugar maple / beech / yellow birch : 53.6% |
| CAR1197 | 86 | 319 | Beech : 33.7%<br>Sugar maple : 28.2%<br>Yellow birch : 10.8% | Sugar maple / beech / yellow birch : 99.8% |
| CAR973 | 37 | 999 | Eastern hemlock : 10.7%<br>Red maple : 25.9%<br>Sugar maple : 28.0% | Sugar maple / beech / yellow birch : 95.6% |
| CAR973 | 39 | 999 | Eastern hemlock : 10.7%<br>Red maple : 25.9%<br>Sugar maple : 28.0% | Sugar maple / beech / yellow birch : 98.3% |
| CAR1066 | 79 | 297 | White fir : 71.8%<br>Red cedar : 13.9% | California mixed conifer : 18.5%<br>White fir : 78.1% |
| CAR1041 | 79 | 297 | Douglas fir : 58.7% | California mixed conifer : 16.0%<br>Douglas fir : 78.0% |
| CAR1104 | 79 | 297 | Douglas fir : 35.6%<br>Tanoak : 28.5%<br>Pacific madrone : 15.0% | Douglas fir : 17.5%<br>Tanoak : 69.9% |
| ACR173 | 79 | 297 | Douglas fir : 56.4%<br>Pacific madrone : 12.1%<br>California black oak : 10.1% | California mixed conifer : 21.5%<br>Douglas fir : 67.2% |
| CAR1191 | 55 | 221 | Douglas fir : 29.4%<br>Redwood : 20.0% | Douglas fir : 11.9%<br>Tanoak : 86.8% |

| | | | | |
|---|---|---|---|---|
| | | | Tanoak : 43.0% | |
| ACR210 | 39 | 151 | Eastern hemlock : 19.4%<br>Northern white-cedar : 46.3% | Northern white-cedar : 79.1% |
| ACR210 | 39 | 152 | Sugar maple : 23.5%<br>Red maple : 12.6%<br>Eastern hemlock : 42.8% | Sugar maple / beech / yellow birch : 89.4% |
| ACR210 | 39 | 153 | Northern red oak : 12.1%<br>Eastern whitepine : 43.6%<br>Red pine : 15.2% | Eastern white pine / northern red oak / white ash : 29.6%<br>Eastern white pine : 64.5% |
| ACR210 | 39 | 154 | Quaking aspen : 10.1%<br>Red maple : 13.4%<br>Eastern hophornbeam : 12.4%<br>Eastern whitepine : 14.0%<br>Aspen : 20.0% | Sugar maple / beech / yellow birch : 12.3%<br>Aspen : 82.1% |
| ACR276 | 2 | 999 | Chestnut oak : 11.0%<br>Red maple : 13.0% | Yellow-poplar / white oak / northern red oak : 21.7%<br>Chestnut oak / black oak / scarlet oak : 25.8%<br>White oak / red oak / hickory : 38.5% |
| ACR276 | 76 | 999 | Chestnut oak : 11.0%<br>Red maple : 13.0% | Yellow-poplar / white oak / northern red oak : 20.6%<br>Chestnut oak / black oak / scarlet oak : 21.1%<br>White oak / red oak / hickory : 44.1% |
| ACR189 | 79 | 297 | Douglas fir : 26.0%<br>Tanoak : 49.0% | Tanoak : 95.9% |
| ACR303 | 95 | 999 | Douglas fir : 11.9%<br>Ponderosa pine : 43.0% | Ponderosa pine : 95.5% |
| ACR303 | 88 | 999 | Douglas fir : 11.9% | Ponderosa pine : 85.2% |

| | | | | |
|---|---|---|---|---|
| | | | Ponderosa pine : 43.0% | |
| CAR1173 | 24 | 999 | Sugar maple : 20.1%<br>Yellow-poplar : 18.7%<br>Chestnut oak : 12.5%<br>White oak : 11.6% | Chestnut oak / black oak / scarlet oak : 10.9%<br>Yellow-poplar / white oak / northern red oak : 37.1%<br>White oak / red oak / hickory : 37.8% |
| CAR1173 | 76 | 999 | Sugar maple : 20.1%<br>Yellow-poplar : 18.7%<br>Chestnut oak : 12.5%<br>White oak : 11.6% | Yellow-poplar / white oak / northern red oak : 34.5%<br>White oak / red oak / hickory : 39.7% |
| CAR1046 | 79 | 999 | Douglas fir : 35.7%<br>White fir : 22.6%<br>Tanoak : 13.9% | California mixed conifer : 37.6%<br>Douglas fir : 49.5% |
| CAR1209 | 35 | 999 | American basswood : 19.3%<br>Sugar maple : 56.1% | Sugar maple / beech / yellow birch : 16.3%<br>Hard maple / basswood : 83.5% |
| CAR1209 | 38 | 999 | American basswood : 19.3%<br>Sugar maple : 56.1% | Sugar maple / beech / yellow birch : 23.5%<br>Hard maple / basswood : 76.4% |
| CAR1257 | 2 | 3 | Chestnut oak : 31.2%<br>Northern red oak : 20.6%<br>Red maple : 20.0% | White oak / red oak / hickory : 18.0%<br>Chestnut oak : 21.9%<br>Chestnut oak / black oak / scarlet oak : 50.1% |
| CAR1257 | 2 | 4 | Chestnut oak : 15.8%<br>Yellow-poplar : 15.6%<br>Red maple : 14.9% | White oak / red oak / hickory : 20.7%<br>Yellow-poplar / white oak / northern red oak : 28.5%<br>Chestnut oak / black oak / scarlet oak : 39.1% |
| CAR1257 | 2 | 7 | Sugar maple : 22.9%<br>Yellow-poplar : 16.0% | Hard maple / basswood : 13.3%<br>Sugar maple / beech / yellow birch : 15.4%<br>Yellow-poplar / white oak / northern red oak : 25.7%<br>White oak / red oak / hickory : 29.8% |

| | | | | |
|---|---|---|---|---|
| CAR1257 | 2 | 8 | Chestnut oak : 21.2%<br>Northern red oak : 12.9%<br>Yellow-poplar : 11.5%<br>Pignut hickory : 11.3%<br>Red maple : 10.2% | Yellow-poplar / white oak / northern red oak : 13.6%<br>White oak / red oak / hickory : 32.8%<br>Chestnut oak / black oak / scarlet oak : 45.4% |
| CAR1257 | 76 | 292 | Sugar maple : 22.9%<br>Yellow-poplar : 16.0% | Hard maple / basswood : 12.4%<br>Sugar maple / beech / yellow birch : 16.9%<br>Yellow-poplar / white oak / northern red oak : 24.9%<br>White oak / red oak / hickory : 30.4% |
| CAR1257 | 76 | 291 | Chestnut oak : 21.2%<br>Northern red oak : 12.9%<br>Yellow-poplar : 11.5%<br>Pignut hickory : 11.3%<br>Red maple : 10.2% | Yellow-poplar / white oak / northern red oak : 14.0%<br>White oak / red oak / hickory : 36.2%<br>Chestnut oak / black oak / scarlet oak : 41.9% |
| ACR284 | 1 | 1 | Eastern hemlock : 43.5%<br>Eastern whitepine : 22.3%<br>Red maple : 12.0% | Sugar maple / beech / yellow birch : 36.3%<br>Eastern white pine / eastern hemlock: 46.1% |
| ACR284 | 1 | 2 | Sugar maple : 20.9%<br>Red maple : 16.8%<br>Beech : 11.8%<br>Eastern whitepine : 10.3%<br>White ash : 10.3% | Sugar maple / beech / yellow birch : 88.8% |
| ACR284 | 41 | 162 | Red maple : 24.3%<br>Northern red oak : 20.3%<br>Eastern whitepine : 17.2%<br>Eastern hemlock : 16.5% | White oak / red oak / hickory : 20.3%<br>Sugar maple / beech / yellow birch : 58.3% |
| ACR284 | 41 | 163 | Eastern hemlock : 43.5%<br>Eastern whitepine : 22.3%<br>Red maple : 12.0% | Eastern white pine / eastern hemlock : 37.6%<br>Sugar maple / beech / yellow birch : 42.3% |

| | | | | |
|---|---|---|---|---|
| ACR284 | 41 | 164 | Sugar maple : 20.9%<br>Red maple : 16.8%<br>Beech : 11.8%<br>Eastern whitepine : 10.3%<br>White ash : 10.3% | Sugar maple / beech / yellow birch : 87.3% |
| CAR1297 | 44 | 999 | Douglas fir : 75.7% | Douglas fir : 96.6% |
| CAR1215 | 2 | 3 | Eastern hemlock : 21.4%<br>Red maple : 17.6%<br>Scarlet oak : 10.1% | Sugar maple / beech / yellow birch : 19.9%<br>White oak / red oak / hickory : 20.7%<br>Chestnut oak / black oak / scarlet oak : 21.7%<br>Yellow-poplar / white oak / northern red oak : 25.3% |
| CAR1215 | 2 | 4 | Yellow-poplar : 19.5%<br>Red maple : 16.1% | Cherry / white ash / yellow-poplar : 12.2%<br>Chestnut oak / black oak / scarlet oak : 16.4%<br>White oak / red oak / hickory : 17.8%<br>Yellow-poplar / white oak / northern red oak : 36.1% |
| CAR1215 | 2 | 7 | Yellow-poplar : 17.6%<br>Sugar maple : 15.8%<br>Red maple : 10.7% | White oak / red oak / hickory : 25.5%<br>Yellow-poplar / white oak / northern red oak : 38.5% |
| CAR1215 | 2 | 8 | Chestnut oak : 33.0%<br>Red maple : 19.2% | Chestnut oak : 31.5%<br>Chestnut oak / black oak / scarlet oak : 58.0% |
| CAR1215 | 2 | 5 | Red maple : 22.6%<br>Yellow-poplar : 19.6%<br>White oak : 12.7% | Chestnut oak / black oak / scarlet oak : 13.3%<br>White oak / red oak / hickory : 25.1%<br>Yellow-poplar / white oak / northern red oak : 40.5% |
| CAR1215 | 24 | 86 | Eastern hemlock : 21.4%<br>Red maple : 17.6%<br>Scarlet oak : 10.1% | White oak / red oak / hickory : 19.5%<br>Chestnut oak / black oak / scarlet oak : 28.8%<br>Yellow-poplar / white oak / northern red oak : 30.9% |

| | | | | |
|---|---|---|---|---|
| CAR1215 | 24 | 87 | Yellow-poplar : 19.5%<br>Red maple : 16.1% | Cherry / white ash / yellow-poplar : 10.0%<br>White oak / red oak / hickory : 18.9%<br>Chestnut oak / black oak / scarlet oak : 19.2%<br>Yellow-poplar / white oak / northern red oak : 35.4% |
| CAR1215 | 24 | 89 | Yellow-poplar : 17.6%<br>Sugar maple : 15.8%<br>Red maple : 10.7% | White oak / red oak / hickory : 29.4%<br>Yellow-poplar / white oak / northern red oak : 38.9% |
| CAR1215 | 24 | 90 | Chestnut oak : 33.0%<br>Red maple : 19.2% | Chestnut oak : 28.9%<br>Chestnut oak / black oak / scarlet oak : 60.6% |
| CAR1215 | 24 | 91 | Red maple : 22.6%<br>Yellow-poplar : 19.6%<br>White oak : 12.7% | Chestnut oak / black oak / scarlet oak : 16.6%<br>White oak / red oak / hickory : 26.8%<br>Yellow-poplar / white oak / northern red oak : 37.9% |
| ACR281 | 38 | 999 | Basswood : 10.8%<br>Red maple : 12.4%<br>Sugar maple : 36.0% | Hard maple / basswood : 19.6%<br>Sugar maple / beech / yellow birch : 79.4% |
| CAR1175 | 94 | 999 | Red spruce : 20.3%<br>Balsam fir : 14.2%<br>Eastern hemlock : 11.6%<br>Red maple : 10.8% | Red spruce : 29.0%<br>Sugar maple / beech / yellow birch : 50.6% |
| CAR1175 | 42 | 999 | Red spruce : 20.3%<br>Balsam fir : 14.2%<br>Eastern hemlock : 11.6%<br>Red maple : 10.8% | Red spruce : 28.4%<br>Sugar maple / beech / yellow birch : 51.3% |
| CAR993 | 55 | 221 | Douglas fir : 30.3%<br>Tanoak : 43.3% | Tanoak : 87.7% |
| CAR993 | 79 | 297 | Douglas fir : 30.3%<br>Tanoak : 43.3% | Tanoak : 91.9% |
| ACR378 | 79 | 297 | Tanoak : 59.6%<br>Douglas fir : 16.6% | Tanoak : 99.1% |

| | | | | |
|---|---|---|---|---|
| ACR377 | 79 | 297 | Tanoak : 45.7%<br>Douglas fir : 33.4% | Tanoak : 90.7% |
| ACR260 | 25 | 999 | Douglas fir : 30.9%<br>Mountain hemlock : 18.8%<br>Grand fir : 13.3% | Douglas fir : 81.8% |
| ACR260 | 58 | 999 | Douglas fir : 30.9%<br>Mountain hemlock : 18.8%<br>Grand fir : 13.3% | Douglas fir : 85.8% |
| ACR371 | 41 | 999 | Eastern whitepine : 32.0%<br>Pin cherry : 16.0%<br>Sassafras : 11.6% | Eastern white pine / northern red oak / white ash : 37.1%<br>Sugar maple / beech / yellow birch : 62.9% |
| CAR1174 | 79 | 297 | Douglas fir : 52.9%<br>Tanoak : 13.2%<br>California black oak : 11.6% | California mixed conifer : 27.6%<br>Douglas fir : 54.0% |
| ACR417 | 42 | 167 | Black spruce : 13.1%<br>Northern white-cedar : 65.7% | |
| ACR417 | 42 | 168 | Red maple : 35.4%<br>Red spruce : 11.0%<br>Yellow birch : 15.9% | Sugar maple / beech / yellow birch : 90.2% |
| ACR417 | 42 | 169 | Beech : 23.7%<br>Sugar maple : 21.8%<br>Yellow birch : 13.6% | Sugar maple / beech / yellow birch : 100.0% |
| ACR417 | 42 | 170 | Eastern hemlock : 12.8%<br>Eastern whitepine : 39.4% | Eastern white pine / eastern hemlock: 12.2%<br>Eastern white pine / northern red oak / white ash : 24.6%<br>Eastern white pine : 55.7% |
| ACR417 | 42 | 171 | Balsam fir : 10.1%<br>Paper birch : 33.7%<br>Quaking aspen : 18.2%<br>Red maple : 20.9% | Paper birch : 90.6% |

| | | | | |
|---|---|---|---|---|
| ACR417 | 42 | 172 | Balsam fir : 27.2%<br>Red spruce : 23.3% | Red spruce : 11.9%<br>Balsam fir : 13.6%<br>Sugar maple / beech / yellow birch : 16.3%<br>Red spruce / balsam fir : 55.4% |
| ACR373 | 38 | 149 | Basswood : 11.7%<br>Red maple : 25.5%<br>Sugar maple : 30.2% | Sugar maple / beech / yellow birch : 92.7% |
| ACR282 | 79 | 297 | Douglas fir : 31.9%<br>Tanoak : 24.6%<br>Redwood : 20.6%<br>Red alder : 11.7% | Tanoak : 40.7%<br>Douglas fir : 49.1% |
| ACR282 | 55 | 221 | Douglas fir : 31.9%<br>Tanoak : 24.6%<br>Redwood : 20.6%<br>Red alder : 11.7% | Douglas fir : 33.9%<br>Tanoak : 58.5% |
| CAR1095 | 79 | 297 | Douglas fir : 41.0%<br>Ponderosa pine : 13.0%<br>California black oak : 13.0%<br>Southern scrub oak : 15.0% | California black oak : 14.8%<br>Douglas fir : 24.6%<br>California mixed conifer : 48.3% |
| ACR255 | 60 | 231 | Ponderosa pine : 29.6%<br>Douglas fir : 52.0% | Ponderosa pine : 17.4%<br>Douglas fir : 82.1% |
| ACR255 | 60 | 233 | Subalpine fir : 12.1%<br>Western larch : 20.9%<br>Lodgepole pine : 22.8%<br>Douglas fir : 29.8% | Engelmann spruce / subalpine fir: 13.3%<br>Lodgepole pine : 14.9%<br>Western larch : 17.9%<br>Douglas fir : 49.0% |
| ACR255 | 22 | 82 | Ponderosa pine : 44.8%<br>Douglas fir : 44.3% | Douglas fir : 43.4%<br>Ponderosa pine : 55.8% |
| CAR1103 | 55 | 221 | Douglas fir : 54.4%<br>Tanoak : 16.6% | Tanoak : 12.4%<br>California mixed conifer : 21.2%<br>Douglas fir : 56.1% |

| | | | | |
|---|---|---|---|---|
| CAR1103 | 79 | 297 | Douglas fir : 54.4%<br>Tanoak : 16.6% | Tanoak : 12.8%<br>California mixed conifer : 17.2%<br>Douglas fir : 60.7% |
| ACR211 | 95 | 353 | Douglas fir : 15.0%<br>Ponderosa pine : 68.0% | Ponderosa pine : 99.0% |
| ACR211 | 95 | 350 | Douglas fir : 25.0%<br>Quaking aspen : 22.0%<br>Ponderosa pine : 13.0%<br>Engelmann spruce : 11.0%<br>White fir : 17.0% | Douglas fir : 90.3% |
| ACR324 | 286 | 286 | Alaska yellow-cedar : 14.1%<br>Sitka spruce : 13.9%<br>Western red cedar : 14.9%<br>Western hemlock : 40.2%<br>Mountain hemlock : 14.3% | Western hemlock : 90.9% |
